# Preliminary Clinical Application of RGD-Containing Peptides as PET Radiotracers for Imaging Tumors

**DOI:** 10.3389/fonc.2022.837952

**Published:** 2022-03-02

**Authors:** Li Li, Xiaoyuan Chen, Jinming Yu, Shuanghu Yuan

**Affiliations:** ^1^ Department of Radiation Oncology, Shandong Cancer Hospital and Institute, Shandong First Medical University and Shandong Academy of Medical Sciences, Shandong Cancer Hospital Affiliated to Shandong First Medical University, Jinan, China; ^2^ Departments of Diagnostic Radiology, Surgery, Chemical and Biomolecular Engineering, and Biomedical Engineering, Yong Loo Lin School of Medicine and Faculty of Engineering, National University of Singapore, Singapore, Singapore; ^3^ Clinical Imaging Research Centre, Centre for Translational Medicine, Yong Loo Lin School of Medicine, National University of Singapore, Singapore, Singapore; ^4^ Nanomedicine Translational Research Program, NUS Center for Nanomedicine, Yong Loo Lin School of Medicine, National University of Singapore, Singapore, Singapore; ^5^ Department of Radiation Oncology, Shandong Cancer Hospital Affiliated to Shandong University, Jinan, China; ^6^ Department of Radiation Oncology, The Affiliated Cancer Hospital of Zhengzhou University, Zhengzhou, China

**Keywords:** RGD, FDG, PET/CT imaging, diagnosis, differential diagnosis, tumor subvolume delineation, therapeutic response prediction

## Abstract

Angiogenesis is a common feature of many physiological processes and pathological conditions. RGD-containing peptides can strongly bind to integrin αvβ3 expressed on endothelial cells in neovessels and several tumor cells with high specificity, making them promising molecular agents for imaging angiogenesis. Although studies of RGD-containing peptides combined with radionuclides, namely, ^18^F, ^64^Cu, and ^68^Ga for positron emission tomography (PET) imaging have shown high spatial resolution and accurate quantification of tracer uptake, only a few of these radiotracers have been successfully translated into clinical use. This review summarizes the RGD-based tracers in terms of accumulation in tumors and adjacent tissues, and comparison with traditional ^18^F-fluorodeoxyglucose (FDG) imaging. The value of RGD-based tracers for diagnosis, differential diagnosis, tumor subvolume delineation, and therapeutic response prediction is mainly discussed. Very low RGD accumulation, in contrast to high FDG metabolism, was found in normal brain tissue, indicating that RGD-based imaging provides an excellent tumor-to-background ratio for improved brain tumor imaging. However, the intensity of the RGD-based tracers is much higher than FDG in normal liver tissue, which could lead to underestimation of primary or metastatic lesions in liver. In multiple studies, RGD-based imaging successfully realized the diagnosis and differential diagnosis of solid tumors and also the prediction of chemoradiotherapy response, providing complementary rather than similar information relative to FDG imaging. Of most interest, baseline RGD uptake values can not only be used to predict the tumor efficacy of antiangiogenic therapy, but also to monitor the occurrence of adverse events in normal organs. This unique dual predictive value in antiangiogenic therapy may be better than that of FDG-based imaging.

## Introduction

Angiogenesis, a well-known process by which new blood vessels sprout from the pre-existing capillaries, includes vascular endothelial cell activation, the degradation of vascular basement membrane during the activation, proliferation and migration of endothelial cells, and the construction of new blood vessels and vascular networks. It is associated with a long list of physiological and pathological events (1, 2). Under normal physiological conditions, such as embryonic development, wound healing, and the menstrual cycle, angiogenesis serves primarily to ensure adequate nutrient and oxygen supply and waste removal. Of course, normal angiogenesis also occurs in pathological conditions, such as tissue damage after reperfusion of ischemic tissue or cardiac failure (3, 4). Unlike the normal angiogenesis described above, both solid and hematologic tumors feature a high dependence on persistent abnormal angiogenesis (5–7). Solid tumors initially form as avascular masses that rely on the vascular system in the host microenvironment, but upon reaching a diameter of 1–2 mm, these tumors exhibit excessive dysfunctional angiogenesis to obtain essential oxygen and nutrients (8–11). The initial recognition of angiogenesis as an interesting process in oncology began in the early 1970s, when Drs. Folkman and Denekamp put forward the idea that highly immature neovascularized tumors are vulnerable via their blood supply. The immature vessels are characterized by an undifferentiated endothelium and a lack of smooth muscles, and act as the vehicles for peripheral sprouting of new capillaries, invasion and distant metastasis (12–15). These mechanisms are facilitated by the synergistic effects of transmission signals from the extracellular matrix (ECM) to endothelial cells, among which integrin binding to endothelial cell recognition sites is the first process to occur. Importantly, the tripeptide sequence of Arg-Gly-Asp (RGD) was identified as a common recognition site in the ECM and blood, and can bind to a variety of adhesion proteins and integrins (16).

The contribution of the RGD tripeptide sequence to cell attachment was first recognized in fibronectin by Pierschbacher and Ruoslahti in 1984 and later confirmed in more ligands, namely, vitronectin, fibrinogen, decorsin, von Willebrand factor, thrombospondin, disintegrins, laminin, entactin, tenascin, prothrombin, osteopontin, bone sialoprotein, adenovirus penton base protein, and collagens (16–18). Combinations of different alpha (α) and beta (β) subunits, such as αvβ3, αvβ5, αvβ8, α5β1 and α8β1, act as receptors for many of the above-mentioned proteins, mainly acting on angiogenesis, inflammation, thrombosis, lung development, cell differentiation, and other pathophysiological processes (19–22). Among these, αvβ3 is highly expressed on angiogenic endothelial cells and several tumor cells, but is expressed only at low levels by resting endothelial cells of normal tissues (23–26). The αvβ3 expression level has also been reported to be a promising factor in tumor diagnosis, staging and therapy (27–29). Due to the high specific affinity between RGD and αvβ3, radiolabeled RGD peptides have been intensively studied for their ability to represent αvβ3 expression density and the tumor angiogenesis state noninvasively (30, 31). Combinations of RGD-containing peptides with radionuclides such as ^18^F, ^64^Cu, and ^68^Ga, have been introduced successfully in positron emission tomography/computed tomography (PET/CT) imaging, among which ^18^F has near ideal decay characteristics for PET (t_1/2_ = 110 min, β^+^ = 0.64 MeV). A linear RGD peptide was first labeled with ^18^F via solid-phase synthesis in 2001, but this radiotracer showed nonspecific accumulation within tumor tissue and early breakdown (32). Then, the cyclic pentapeptide cyclo (Arg-Gly-Asp-D-Phe-Val) (denoted as cyclic RGD peptide) was prepared and found to be a highly potent, stable and selective inhibitor of integrin signaling (33, 34). In addition, based on the polyvalency effect, different types of dimeric and multimeric cyclic RGD peptides were prepared to improve αvβ3-binding affinities for intense tumor accumulation and increased tumor/background ratios compared with the monomeric compounds (35–37). During the optimization of radiotracers, strategies such as cyclization, glycosylation, pegylation, multimerization, and insertion of spacers/linkers have been adopted to optimize the in vitro and in vivo behaviors of radiolabeled RGD peptides (38–41). However, few of these RDG peptide labeling methods have been successfully translated from the bench to clinical use.

Therefore, in this review, we focus on the preliminary clinical applications of PET radionuclide-conjugated RGD-containing peptides that target RGD-binding integrins (mainly αvβ3 integrin), discussing their distribution characteristics and potential use in diagnosis, delineation of tumor subvolume, evaluation response of chemoradiotherapy or antiangiogenic therapy, and the comparative results achieved with traditional ^18^F-fluorodeoxyglucose (^18^F-FDG) imaging agent in the context of functional tumor imaging.

## Comparative Characteristics of RGD-Based and FDG Tracers in Clinical Application

**Figure 1:**
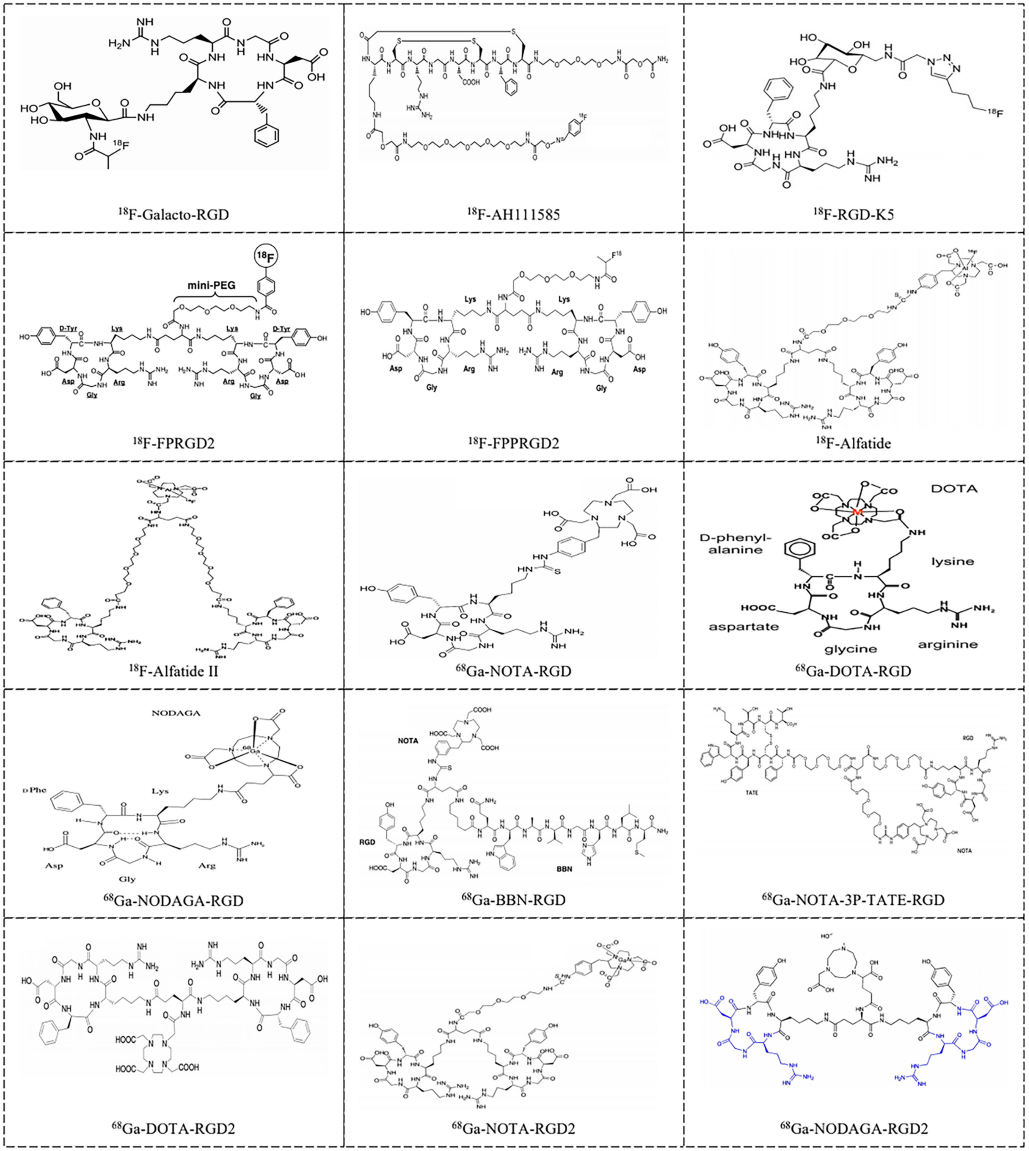
Examples of RGD-based radiotracers [^18^F-Galacto-RGD (42), ^18^F-AH111585 (42), ^18^F-RGD-K5 (43), ^18^F-FPRGD2 (44), ^18^F-FPPRGD2 (45), ^18^F-Alfatide (46), ^18^F-Alfatide II (47), ^68^Ga-NOTA-RGD (48), ^68^Ga-DOTA-RGD (49), ^68^Ga-NODAGA-RGD (50), ^68^Ga-BBN-RGD (51), ^68^Ga-NOTA-3P-TATE-RGD (52), ^68^Ga-DOTA-RGD2 (53), ^68^Ga-NOTA-PRGD2 (54) and ^68^Ga-NODAGA-RGD2 (55)] for imaging tumor angiogenesis in clinical application.

Monomeric and multimeric RGD-based PET tracers that have been investigated in preliminary clinical studies are listed in **Supplementary Table 1** and **Figure 1**. ^18^F-Galacto-RGD, which is a glycosylated RGD-peptide, was initially reported by Haubner et al. in 2005 and was the first monomeric integrin-specific PET tracer used in patients (56). It is well tolerated, as all studies reported neither drug-related adverse events nor important clinical indicators. The dose distribution results were consistent for a variety of tumor types, with similar evidence of predominant renal excretion, fast blood elimination, rapid tumor tracer accumulation, and low background concentration in most organs, namely, the lung, muscle tissue and brain, allowing imaging of tumor lesions with good contrast (56–58). A relatively small amount of ^18^F-Galacto-RGD is also excreted via the hepatobiliary pathway, and thus, in some patients, tracer uptake is seen in the gallbladder. Similar biodistribution results were observed for the other monomeric radionuclide tracers such as ^18^F-fluciclatide (denoted as ^18^F-AH111585) (59–61) and ^18^F-RGD-K5 (43). Consistent with the polyvalency effect caused by dimerization and multimerization of cyclic RGD peptides, series of RGD radiotracers were found to show improved radiosynthesis yields, relatively higher tumor integrin-specific accumulation and favorable in vivo pharmacokinetic properties compared with monomeric ones in both preclinical and clinical studies (44, 45, 59, 62–68). However, the multistep, time-consuming and low-yield synthetic procedures make an automated production process, which is mandatory for routine clinical use, extremely difficult, and thus, the commercial clinical viability of this tracer is limited. Chen et al. successfully established a simplified labeling procedure for ^18^F-fluoride-aluminum complex-labeled RGD tracer ^18^F-AlF-NOTA-PRGD2 (denoted as ^18^F-Alfatide) and ^18^F-AlF-NOTA-E[PEG4-c(RGDfk)]2 (denoted as ^18^F-Alfatide II) in one single step of radiosynthesis (46, 54, 69). Upon successful introduction of a simple, one-step, lyophilized kit for ^18^F-alfatide preparation, with which complete radiosynthesis including purification can be accomplished within 30 min with a radiochemical purity of more than 95%, the potential for clinical transformation of this radiotracer was greatly improved. The pharmacokinetics and imaging properties of ^18^F-Alfatide were comparable to those of ^18^F-FP-PEG3-E[c(RGDyK)]2 (denoted as ^18^F-FPPRGD2) (54, 70). In addition, the positive effect of cyclic mono or multimeric peptides was also successfully developed into ^68^Ga-labeled RGD analogs, which have shown promise for imaging integrin αvβ3 expression (48, 71–79).

Unlike the phenomenon of decreasing standard uptake values (SUVs) in normal organs over time, tumor retention of RGD-based PET tracers did not stop increasing until a plateau period was reached, which showed no obvious fluctuation within nearly 40–60 min after tracer injection. It appeared as either a distinct increase in primary tumors and lung, pleura, bone, and lymph node metastases compared with surrounding normal tissue or as moderate or reduced accumulation in the cases of kidneys, bladder, liver, spleen, and intestine. Sixty minutes after injection was recommended as the appropriate imaging time for optimal lesion-to-background contrast, according to the data from RGD-based fluorine-labeled monomeric or dimeric radiotracer studies (44, 45, 57, 59, 60). For biodistribution in normal tissues, RGD tracers can be used to image brain tumors or brain metastases with a clear background, due to their inability to pass through the healthy blood–brain barrier (BBB) but ability to cross the damaged BBB created by the tumor (43, 46, 61). Primary high uptakes were shown in organs such as the kidneys, bladder, liver, spleen and intestine organs, along with diffuse uptake in the bilateral ventricles and thyroid. High uptake in the kidneys and bladder resulted predominantly from urinary excretion. The uptake values of liver and spleen were thought to be physiological, high liver background may result from the densely vascularized nature of the organ and metabolism of RGD-based imaging tracers by the liver (60). Prominent accumulation of RGD peptide ligands in the liver reported in mice and humans also supported this assertion (80, 81). The uptake values in the intestine maybe associated with physiological expression of αvβ3 on intestinal smooth muscle cells, because the uptake of these tracers did not change significantly during an observation period of 90 min (57, 58, 82). While an initial ^18^F-FPPRGD2 increase followed by a pattern of decreasing uptake in the intestine was also observed, this may reflect initial dimeric RGD accumulation was cleared from hepatobiliary excretion (45, 83). Overall, this finding indicates the unsuitability of RGD imaging for hepatocellular carcinoma (HCC) tumors or diseases with liver metastases.

In addition to integrin αvβ3 expression, glucose metabolism is also believed to correlate with tumor aggressiveness and progression (84, 85). In tumor lesions, RGD-based tracer uptake is significantly lower than that of FDG, as commonly observed in studies comparing FDG and RGD tracers (52, 63, 65, 66, 86–92), but this difference was altered in some cases, such as with the higher maximum SUV (SUV_max_) and tumor-to-blood ratio (TBR) of ^68^Ga-NOTA-3P-TATE-RGD compared with ^18^F-FDG in neuroendocrine tumor (NET) cases and also in both human epidermal growth factor receptor 2 negative [HER2 (–)] and estrogen receptor positive [ER (+)] breast cancer (52, 88). One possible reason responsible for this difference may be related to the different binding mechanisms of the tracers. ^18^F-FDG mainly depends on the hypermetabolism of glucose in malignant tumor cells, whereas RGD PET imaging predominantly binds to avβ3 highly expressed in tumor neovascular endothelial cells. Also, within each lesion, the number of endothelial cells expressing integrin αvβ3 is smaller than the abundant number of tumor cells containing glucose transporters (93). In addition, both tracers showed strong uptake in inflammatory lesions similar to malignancies. RGD was thought to be more beneficial than FDG for differentiating inflammation, as it demonstrated significantly lower retention than FDG after comparable high uptake levels in inflammation (56, 94–96). With regard to the correlation between RGD and FDG data, inconsistent results were observed when tumor lesions overall were considered. Moderate or significant correlations between RGD and FDG parameters were reported in locally advanced rectal cancer (LARC) and squamous cell carcinoma of head and neck (HNSCC) (87, 88, 90, 97), and for subgroups of tumors, namely, non-small cell lung cancer (NSCLC), triple-negative breast cancer (TNBC), and FDG-avid tumors (86, 89). However, other studies failed to demonstrate a correlation between RGD and FDG uptake (65, 91, 98–100).

These results overall may indicate the coexistence of interrelated pathophysiological phenomena within the tumor, such as cell proliferation and neoangiogenesis, but the lack of a significant correlation between RGD and FDG uptake suggests that these imaging modalities provide complementary rather than similar information (101). RGD imaging offers indispensable value in terms of evaluating tumor angiogenesis and planning αvβ3-based therapy. As the next logical step, many research groups focused on exploring the clinical characteristics of RGD-based imaging agents.

## Preliminary Diagnostic Value of RGD-Containing Peptide Tracers

In a pan-cancer study with 9 patients (5 with malignant melanomas, 2 with sarcomas, 1 with osseous metastasis from renal cell carcinoma [RCC], and 1 with villonodular synovitis), ^18^F-Galacto-RGD SUVs showed inter- and intra-individual variances ranging from 1.2 to 10.0 (56). Consistently, Beer et al. reported the heterogeneous accumulation of ^18^F-Galacto-RGD from 1.2 to 9.0 in 19 patients. Distribution volume values for tumor tissue (1.5 ± 0.8) were 4 times higher than those for muscle tissue (0.4 ± 0.1), suggesting intense specific receptor binding in tumor tissues and only minimal free and bound (specific or nonspecific) tracer in muscle tissue (57). Similarly, studies of other imaging agents such as ^18^F-AH111585 and ^68^Ga-NODAGA-E[(cRGDyK)_2_] (denoted as ^68^Ga-NODAGA-RGD2) also reported heterogeneous tracer uptake in tumor and metastatic sites and superior tumor-to-background ratios in patients with malignant disease (58, 61, 76, 86, 102). However, the sensitivity of ^18^F-Galacto-RGD (76%) for lesion identification was lower than that of conventional staging with contrast-enhanced CT and ^18^F-FDG PET in a comparative study (86). This is not surprising because, unlike the glycolytic effect of entire tumor cell, αvβ3 is expressed at elevated levels on angiogenic blood vessels and on malignant tumors (103, 104). Interestingly, the related research demonstrated high inter- and intraindividual variances in tracer accumulation in different types of lesions, indicating great diversity in receptor expression.

According to the literature, the expression patterns of αvβ3 are specific on malignant carcinomas and benign tumors or inflammatory tissues (95, 105, 106). In benign lesions (e.g., pigmented villonodular synovitis, neurofibroma, and inflammatory tissue), αvβ3 expression is located only on the vasculature. However, in malignant lesions, αvβ3 is expressed not only on the endothelial cells of neovasculature but also on the surface of some tumor cells. In HNSCC, αvβ3 is located predominantly on the neovasculature but shows some low expression on tumor cells (56, 102, 107). Immunohistochemical analyses confirmed αvβ3 expression predominantly on microvessels (5/5) and, to a lesser extent, on tumor cells (3/5) in invasive ductal breast cancer (IDC) (28, 108). In malignant melanoma and clear cell RCC (ccRCC), αvβ3 is highly located on the surface of tumor cells (61, 102, 109, 110). In the other solid tumors, namely, glioma, lung cancer, differentiated thyroid cancer, and breast cancer (56, 102, 108–112), αvβ3 expression is located on the neovasculature and on the tumor cells at varying degrees. For example, a significant correlation between αvβ3 expression and radiotracer uptake was reported by a study employing ^18^F-Galacto-RGD PET in melanoma and sarcoma patients (56). Specially, in ccRCC cases, the ^18^F-FB-mini-PEG-E[c(RGDyK)]2 (denoted as 18F-FPRGD2) PET signal correlated with integrin αvβ3 expression on tumor cells, and immunohistochemistry results confirmed high expression of αvβ3 on ccRCC cells. In contrast, in papillary RCC (pRCC) cases, the ^18^F-FPRGD2 PET signal correlated with the integrin αvβ3 level on tumor vessels (67). These results demonstrate that RGD-based tracer uptake can reflect integrin αvβ3 expression in renal tumors, but represents angiogenesis only when tumor cells do not express integrin αvβ3. In other words, RGD-based PET may be regarded as a surrogate marker of angiogenesis for the visualization and noninvasive quantitation of integrin expression when αvβ3 expression is predominantly confined to the tumor vasculature as in HNSCC (107). Overall, the association between RGD SUVs and TBR and the corresponding αvβ3 expression level demonstrated the value of noninvasive techniques for appropriately diagnosing tumor lesions, although a positive correlation was not always clear (56, 61, 107, 113–116). The heterogeneous distribution pattern among different lesions described above may explain this phenomenon. Next, we provide a review of reported clinical investigations related to the application of RGD-based tracers for diagnosis, definition of tumor subvolume, and therapeutic response prediction according to specific tumor types (**Table 1**).

**Table 1:** Preliminary case-by-case diagnostic value of RGD-containing peptides and FDG.

Tumor type	Purpose	Preferred agents	Supporting evidence	Opposing evidence	Reference
GBM	HGG vs. LGG	RGD	^68^Ga-PRGD2: TBR 0.80–2.00 vs. 3.55–11.00, sensitivity 100% (8/8), specificity 100% (4/4)		(114)
			^18^F-FDG: TBR 0.57–1.38 vs. 1.11–4.77; sensitivity 88% (7/8), specificity 75% (3/4)		
	meningioma vs. HGG	RGD	^68^Ga-NOTA-PRGD2: SUV_max_ 4.23 ± 2.48 vs. 1.57 ± 0.33, p = 0.0047		(115)
			^18^F-FDG: SUV_max_ 5.38 ± 2.37 vs. 12.64 ± 1.91, p = 0.0537		
HNSCC	diagnosis of lesions	RGD and FDG	^68^Ga-NODAGA-RGD vs. ^18^F-FDG: both detected 100% of primary lesions, lymph nodes, distant metastases		(87)
Thyroid cancer	diagnosis of radioiodine-refractory thyroid cancer	RGD	^68^Ga-DOTA-RGD2: sensitivity 82.3%, specificity 100%, accuracy 86.4%, PPV 100%, NPV 62.5%	^68^Ga-DOTA-RGD2: detect 123 lesions. 63.6% patients; 10.6% thyroid bed lesions; 82.9% nodal lesions; 6.5% skeletal lesions	(92)
			^18^F-FDG: sensitivity 82.3%, specificity 50%, accuracy 75%, PPV 84.8%, NPV 45.4%	^18^F-FDG: detect 144 lesions, 75.0% patients; 8.3% thyroid bed lesions; 86.1% nodal lesions; 5.5% skeletal lesions	
	radioiodine-refractory lesions vs. benign lesions	none	^18^F-Alfatide: SUV_mean_ 2.5 ± 0.9 vs. 2.8 ± 0.9, p = 0.576		(91)
			^18^F-FDG: SUV_mean_ 4.5 ± 1.6 vs. 3.3 ± 1.2, p = 0.133		
	radioiodine-refractory lesions vs. iodine-avid lesions	none	^18^F-Alfatide: SUV_mean_ p >0.05		
			^18^F-FDG: SUV_mean_ p >0.05		
	lesions diameter >1.5 cm vs. <1.5 cm in differentiated thyroid cancer	RGD	^18^F-Alfatide: SUV_mean_ 3.1 ± 0.7 vs. 2.4 ± 0.8, p <0.05		
			^18^F-FDG: SUV_mean_ p >0.05		
	lymph nodes diameter >1 cm vs. <1 cm in differentiated thyroid cancer	none	^18^F-Alfatide: SUV_mean_ p = 0.06; ^18^F-FDG: SUV_mean_ p = 0.41		
Lung cancer	malignancies vs. benign ones	undefined	^68^Ga-NOTA-PRGD2: sensitivity 83.8%, specificity 91.3%, accuracy 85.7% (SUV_mean_ = 1.3); sensitivity 80.9%, specificity 82.6%, accuracy 81.3% (SUV_max_ = 2.0)	^18^F-FDG: sensitivity 86.8%, specificity 69.6%, accuracy 82.4% (SUV_mean_ = 2.0); sensitivity 85.3%, specificity 69.6%, accuracy 81.3% (SUV_max_ = 3.0)	(100)
	metastatic vs. non-metastatic lymph nodes	RGD	^68^Ga-NOTA-PRGD2: SUV_max_ 1.93 ± 1.03 vs. 0.75 ± 0.75, p <0.05; PPV 90.0%, NPV 93.8%		(100)
			^18^F-FDG: SUV_max_ 3.91 ± 2.37 vs. 2.30 ± 2.31, p = 0.48; PPV 30.2%, NPV 90.5%		
			^18^F-FDG: sensitivity 75%, specificity 66.7%		
	lung cancer vs. tuberculosis	RGD	^18^F-Alfatide II: SUV_max_ 4.08 ± 1.51 vs. 2.63 ± 1.34, p = 0.0078		(117)
			^18^F-FDG: SUV_max_ 12.04 ± 4.67 vs. 7.53 ± 2.88		
	NSCLC vs. SCLC	RGD	^68^Ga-DOTA-RGD2: SUV_max_ 3.83 ± 1.00 vs. 2.05 ± 0.74, p <0.0001; TNR 1.94 ± 0.70 vs. 0.94 ± 0.24, p = 0.0099		(111)
			^18^F-FDG: SUV_max_ 10.39 ± 4.03 vs. 7.84 ± 2.25, p = 0.08;TNR 5.03 ± 2.52 vs. 3.62 ± 0.93, p = 0.10		
	diagnosis of neuroendocrine tumor	RGD	^68^Ga-NOTA-3P-TATE-RGD vs. ^18^F-FDG: 24.12 ± 22.39 vs. 3.75 ± 2.81, p = 0.0241		(52)
ESCC	pathological stages I–II vs. III–IV	RGD and FDG	^18^F-Alfatide: 2.41 ± 1.06 vs. 3.41 ± 0.68, p = 0.03		
			^18^F-FDG: 4.02 ± 1.83 vs. 7.24 ± 3.70, p = 0.002		
	positive vs. negative lymph nodes	RGD and FDG	^18^F-Alfatide: 3.57 ± 0.58 vs. 2.16 ± 0.80, p = 0.001		
			^18^F-FDG: 7.41 ± 3.35 vs. 3.2 ± 0.44, p < 0.001		(118)
	well vs. moderate vs. poor differentiation	RGD	^18^F-Alfatide: 2.44 ± 0.83 vs. 2.91 ± 0.62 vs. 3.56 ± 0.98, p = 0.049		
			^18^F-FDG: 3.26 ± 0.36 vs. 4.49 ± 1.67 vs. 7.05 ± 4.02, p = 0.053		
Breast cancer	diagnosis of breast and metastatic lesions	RGD	^18^F-FPPRGD2: sensitivity, all lesions 95.7%, breast lesions 100%, axillary lymph nodes 75.0%, bone 100%, other metastases 100%; specificity, all lesions 100%, breast lesions 100%, axillary lymph nodes 100%, bone 100%, other metastases 100%	^18^F-RGD-K5 detected only 122 lesions (77.7%) among 157 lesions observed on ^18^F-FDG PET	(98)
			^18^F-FDG: sensitivity, all lesions 87.0%, breast lesions 83.3%, axillary lymph nodes 75.0%, bone 87.5%, other metastases 100%; specificity, all lesions 57.1%, breast lesions 100%, axillary lymph nodes 0%, bone 100%, other metastases 0%		(63)
	breast cancer vs. benign breast lesion	undefined	^18^F-Alfatide II: sensitivity 97.6% (SUV_max_ = 1.6); ^18^F-FDG: sensitivity 78.6% (SUV_max_ = 3.68)	^18^F-Alfatide II: specificity 54.5%, Youden index 52.1%, AUC 0.738; ^18^F-FDG: specificity 81.8%, Youden index 60.4%, AUC 0.838	(88)
	different HR status	RGD	^68^Ga-BBN-RGD: ER(+) vs. ER (–), p = 0.0083 ^18^F-Alfatide II: Luminal A/Luminal B vs. Triple negative, p = 0.032/0.026; ^68^Ga-NOTA-RGD: ER/PR(+),HER2(−) or ER/PR(+),HER2(+) vs. ER/PR(−),HER2(−), p <0.05	^18^F-FDG: ER/PR(+),HER2(−) or ER/PR(+),HER2(+) vs. ER/PR(−),HER2(−), p <0.05	(116)
	different HER2 status	FDG	^18^F-FDG: HER-2(+) vs. Luminal A/Luminal B, p = 0.001/0.000 ^68^Ga-BBN-RGD: HER-2(+) vs. HER-2(−), p = 0.6589	^68^Ga-NOTA-RGD: HER2(+) vs. HER2(−), 2.90 ± 0.75 vs. 2.42 ± 0.59, p = 0.04 for SUV_max_; 1.95 ± 0.53 vs. 1.60 ± 0.38, p = 0.04 for SUV_mean_	(89)
HCC or liver metastasis	diagnosis	FDG	^68^Ga-DOTA RGD: 0 patients; ^18^F-FDG: detect liver metastases in 9 patients (16%)		(119)
LARC	diagnosis	FDG	^18^F-FPRGD2: 18 (51%) pelvic lymph nodes; ^18^F-FDG: 35 pelvic lymph nodes		(66)
Metastatic RCC	diagnosis	undefined	^18^F-FPPRGD2: 55/60 lesions; ^18^F-FDG: 52/60 lesions		(97)
Cervical cancer and ovarian cancer	diagnosis	undefined	^18^F-FPPRGD2: 48/52 lesions; ^18^F-FDG: 50/52 lesions		(90)
Brain metastasis	diagnosis	RGD	^18^F-Alfatide II: detected 100% of lesions, TNR 18.9 ± 14.1; ^18^F-FDG: 50% of lesions, TNR 1.5 ± 0.5		(47)
Bone metastasis	diagnosis	undefined	^18^F-Alfatide II: 100% of osteolytic metastases, 70% of osteoblastic metastases, 100% of mixed bone metastases, 98% of bone marrow metastases, 92% of all metastases		(120)
			^18^F-FDG: 90% of osteolytic metastases, 53% of osteoblastic metastases, 90% of mixed bone metastases, 77% of bone marrow metastases, 77% of all metastases		

GBM, glioblastoma multiforme; HGG, high-grade glioma; LGG, low-grade glioma; TBR, tumor-to-blood ratio; SUV_max_, maximum standard uptake values; HNSCC, squamous cell carcinoma of head and neck; PPV, positive predictive value; NPV, negative predictive value; NSCLC, non-small cell lung cancer; SCLC, small cell lung cancer; TNR, tumor-to-normal ratio; ESCC, esophageal squamous cell carcinoma; ER, estrogen receptor; PR, progesterone receptor; HER2, human epidermal growth factor receptor 2; HCC, hepatocellular carcinoma; LARC, locally advanced rectal cancer; RCC, Renal cell cancer.

### Glioblastoma

Tumor tracer uptake was heterogeneously restricted to the periphery of the tumor in glioma patients, with no activity in the necrotic center of the lesions and normal brain tissue. The correlation between tracer accumulation and immunohistochemical staining intensity for αvβ3 expression in 21 tissue samples was weak but significant (r = 0.463, p = 0.034), which provided evidence that ^18^F-Galacto-RGD PET is a promising tool for noninvasively monitoring αvβ3 integrin expression in patients with malignant gliomas (113). However, the characteristics revealed in this study mainly related to diagnosed glioblastoma multiforme (GBM) and recurrent GBM after external beam radiation or chemotherapy, which may reduce the reliability of the results. Li et al. further explored patients with newly diagnosed GBM of different grades in a prospective ^68^Ga-NOTA-PRGD2 (denoted as ^68^Ga-PRGD2) PET/CT imaging study. The results from 12 GBM patients also showed significant accumulation in the tumors, but not in the white matter or cortical gray matter, with the exception of the choroid plexus. The maximum TBR (TBR_max_) of ^68^Ga-PRGD2 PET/CT was superior for differentiating high-grade glioma (HGG) from low-grade glioma (LGG) compared with ^18^F-FDG PET/CT (sensitivity, 100% vs. 88%; specificity, 100% vs. 75%). This clinical case-by-case evaluation provides a sufficient basis for applying RGD-based imaging to determine the boundary and stage of glioma (114). Data obtained by Liu et al. also supported this phenomenon of HGG exhibiting a more intense ^18^F-Alfatide SUV_max_, mean SUV (SUV_mean_), and tumor-to-normal ratio (TNR) than LGG (121). These results could be explained by the higher αvβ3 expression level in HGG compared with LGG (122). Overall, this research demonstrates that RGD-based tracer imaging might be superior to ^18^F-FDG PET/CT for GBM imaging, as the tracer molecules only specifically accumulated in the tumors, while not accumulating in the normal brain tissue as characterized by high ^18^F-FDG uptake.

For distinguishing meningioma and HGG lesions, RGD also provided considerable added value to ^18^F-FDG PET/CT imaging. Li et al. showed that higher SUV_max_ (4.23 ± 2.48) of ^68^Ga-PRGD2 was observed in the uncommon meningiomas than in the HGG lesions (1.57 ± 0.33, p = 0.0047). The uptake ratios of ^68^Ga-PRGD2 over ^18^F-FDG normalized as lg100 ∗ SUV_max_ (RGD/FDG) were significantly higher in the uncommon meningiomas than those in HGG (1.87 ± 1.36 vs. 1.04 ± 0.87, p = 0.0001) with a defined cutoff value of 1.58. However, no significant differences were detected by ^18^F-FDG PET between the rare subtypes of meningioma and HGG (115).

### Head and Neck Squamous Cell Carcinoma (HNSCC)

Given the predominant αvβ3 expression on the neovasculature in HNSCC, angiogenesis maybe the optimal surrogate variable in such cases (56, 110). Eleven patients with HNSCC were examined by ^18^F-Galacto-RGD static emissions scanning, it successfully identified 83.3% (10/12) of tumor-bearing cases and 33.3% (2/6) of pathological metastatic lymph nodes (107). When comparing with ^18^F-FDG uptake, Durante et al. demonstrated that all primary tumors, lymph nodes and distant metastases could be seen using both tracers, with higher tumor uptake was observed in ^18^F-FDG than in ^68^Ga-NODAGA-c(RGDyK) (^68^Ga-NODAGA-RGD) imaging. Notably, the SUV_mean_ for RGD showed a positive correlation with that for ^18^F-FDG (Spearman’s r = 0.89, p = 0.0068) (87). The feasibility of ^68^Ga-DOTA-E-[c(RGDfK)]2 (^68^Ga-DOTA-RGD2) PET/CT for visualizing angiogenesis was also validated in patients with oral squamous cell carcinoma with SUV_max_ ranging from 4.0 to 12.7 (77).

### Thyroid Cancer

In radioiodine refractoryIn differentiated thyroid cancer (RAIR-DTC) patients, ^18^F-FDG PET/CT showed higher SUV_max_ compared to RGD PET/CT whether in primary tumor lesions or lymph node metastasis (91, 92). These two tracers showed similar sensitivities of 82.3% in detecting lesions, but ^68^Ga-DOTA-RGD2 PET/CT had a higher specificity and accuracy of 100 and 86.4% compared to 50 and 75% for ^18^F-FDG PET/CT (92). No significant differences were observed between RAIR lesions and benign lesions by ^18^F-Alfatide and ^18^F-FDG imaging. Notably, ^18^F-Alfatide uptake was significantly higher in lesions with a diameter larger than 1.5 cm than lesions smaller than 1.5 cm (91). These results supported feasibility of RGD PET/CT not only on the diagnosis of RAIR-DTC but also on the evaluation of treatment response of ^177^Lu-DOTA-RGD in these patients, especially in cases deemed negative/suspicious on ^18^F-FDG PET/CT (123).

### Lung Cancer

In lung cancer, RGD has been widely explored in tumor diagnosis and differential diagnosis. ^18^F-Alfatide PET imaging successfully identified all tumors with desirable image contrast, indicating a lower variance in tumor SUV_mean_ (2.90 ± 0.10) and high tumor-to-background ratio [tumor-to-muscle ratio (TMR), 5.87 ± 2.02; TBR, 2.71 ± 0.92] in 9 lung cancer patients ^58^. Later, Gao et al. and Zhou et al. further explored the value of this imaging method in the diagnosis of lymph nodes. Their data showed that the sensitivity, specificity, accuracy, positive predictive value (PPV) and negative predictive value (NPV) were 100, 44.44, 80.77, 77.27, and 100, respectively, for the diagnosis of suspected lung cancer and 100.0, 94.9, 95.4, 69.0, and 100%, respectively, for confirming malignant lymph node metastasis. Significantly higher ^18^F-Alfatide RGD uptake was observed either in differentiating malignant tumor lesions (malignant lesions vs. hamartomas, 5.37 ± 2.17 vs. 1.60 ± 0.11, p <0.001; TBR, 4.13 ± 0.91 vs. 1.56 ± 0.24, p <0.001) or positive lymph node metastases. However, no significant differences were observed between malignant lesions and inflammatory lesions or inflammatory pseudotumors (p >0.05) (124, 125).

In another study using ^68^Ga-NOTA-PRGD2 PET/CT, benign and malignant tumors and lymph nodes were successfully identified in 91 patients with suspected lung lesions. Both the SUV_mean_ and SUV_max_ of primary malignancies were higher than those of benign lesions, and the SUV_max_ of metastatic lymph nodes was higher than that of non-metastatic nodes. In addition, comparative results of ^68^Ga-NOTA-PRGD2 and ^18^F-FDG PET/CT were reported. For ^68^Ga-NOTA-PRGD2, with a cut-off SUV_mean_ of 1.3, malignant lesions could be distinguished from benign lesions with a sensitivity, specificity, and accuracy of 83.8% (57/68), 91.3% (21/23), and 85.7% (78/91), respectively. With a SUV_max_ threshold of 2.0, the corresponding sensitivity, specificity and accuracy were 80.9% (55/68), 82.6% (19/23), and 81.3% (74/91), respectively. By comparison, for ^18^F-FDG PET/CT, with a cut-off SUV_mean_ of 2.0, the sensitivity, specificity, and accuracy were 86.8% (59/68), 69.6% (16/23), and 82.4% (75/91), respectively, and with the SUV_max_ cut-off of 3.0, the sensitivity, specificity, and accuracy of ^18^F-FDG PET/CT for the diagnosis of lung cancer were 85.3% (58/68), 69.6% (16/23), and 81.3% (74/91), respectively. However, for the assessment of lymph node metastasis, the PPV and NPV of ^68^Ga-NOTA-PRGD2 were 90.0% (27/30) and 93.8% (121/129), respectively, compared with 30.2% (29/96) and 90.5% (57/63), respectively for ^18^F-FDG. From these results, ^68^Ga-NOTA-PRGD2 PET/CT offered similar sensitivity and higher specificity for detecting lung lesions compared with ^18^F-FDG PET/CT, but remarkable improvement for the assessment of lymph nodes metastasis with a higher specificity (100, 126).

Consistent results were also obtained for the ability of RGD-based radiotracers to differentiate tuberculosis and lung cancer, with studies reporting that tracer uptake values for ^18^F-Alfatide II was significantly higher in malignant lesions than in tuberculosis, which indicated that RGD-based PET/CT might differentiate lung cancer from tuberculosis (117). The ^68^Ga-NOTA-RGD SUV_max_ allowed for significant delineation of a solitary pulmonary nodule from non-malignant lung tissue (3.7 ± 0.7 vs. 0.4–0.9, p <0.001) (60). Consistent with the different αvβ3 expression patterns in NSCLC and small cell lung cancer (SCLC), NSCLC samples showed positive αvβ3 expression in both tumor cells and the vasculature epithelium, whereas SCLC exhibited inconspicuous αvβ3 expression. Tracer accumulation of ^68^Ga-DOTA-RGD2 was higher in NSCLC (ranging from 5.7 to 1.2) than in SCLC (3.3 to 1.1) for both primary lesions and metastatic lymph nodes. In contrast, synchronous ^18^F-FDG PET/CT examination did not show the differences between NSCLC and SCLC groups (111). ^18^F-Alfatide imaging also showed statistically higher peak SUV (SUV_peak_), SUV_mean_ and total lesion angiogenesis (TLA) for squamous cell carcinoma than for adenocarcinoma (127). Given the low levels of αvβ3 expression in SCLC, ^68^Ga-NOTA-3P-TATE-RGD was developed to overcome the insufficiencies of TATE imaging in NSCLC and RGD imaging in SCLC. The TBR with ^68^Ga-NOTA-3P-TATE-RGD was significantly higher than that with ^68^Ga-NOTA-RGD (6.06 ± 6.09 vs. 2.65 ± 1.19, p = 0.0344) in SCLC, or that with ^18^F-FDG in neuroendocrine tumors (2.91 ± 1.71, p = 0.0234) (52). It is important to note that lung cancer and inflammatory lesions or inflammatory pseudotumors cannot be distinguished with RGD imaging, nor with FDG imaging (124). Based on these data, RGD-related imaging may be an appropriate strategy for αvβ3-associated therapy and imaging, which showed advantages on distinguishing between NSCLC and SCLC.

### Esophageal Cancer

In a case report for a moderately differentiated carcinoma of the gastro-esophageal junction, ^68^Ga-NODAGA-RGD tracer uptake was clearly localized in non-FDG-avid perilesional structures, demonstrating that angiogenesis imaging might be a crucial tool for early disease identification and localization, metastasis evaluation, and efficacy prediction (128). Dong et al. prospectively enrolled 46 patients with newly diagnosed esophageal squamous cell carcinoma (ESCC) (n = 21 for ^18^F-Alfatide PET/CT and n = 25 for ^18^F-FDG PET/CT). The lymph nodes showed different accumulation patterns for separate pathological stages and metastatic status both on ^18^F-Alfatide PET/CT (stages I–II vs. III–IV, p = 0.03; positive vs. negative, p = 0.003) and ^18^F-FDG PET/CT (stages I–II vs. III–IV, p = 0.001. positive vs. negative, p <0.001). These results suggest that tracer uptake characteristics were consistent between ^18^F-Alfatide and ^18^F-FDG PET/CT imaging, and thus, the ^18^F-Alfatide PET SUV in lymph nodes may provide complementary molecular information for risk stratification of ESCC patients (118).

### Breast Cancer

The RGD accumulation was thought to represent the sum of uptake in neovasculature and tumor cells according to the immunohistochemical staining in IDC patients (108). ^18^F-AH111585 tracer uptake either homogeneously distributed in tumors or appearing within the tumor rim in metastatic breast cancer patients, which was consistent with the general principles of angiogenesis localization within the outer zones of breast tumors, where viable tumors exist (129–132). Vatsa et al. reported the results of ^68^Ga-DOTA-RGD2 PET/CT in patients with locally advanced breast carcinoma (LABC) and found both ^68^Ga-DOTA-RGD2 and ^18^F-FDG were similarly able to diagnose primary and metastatic lymph nodes, and RGD-based imaging showed additional value for distinguishing nonspecific ^18^F-FDG uptake due to an infectious or inflammatory etiology (133). The sensitivity and specificity of ^18^F-FPPRGD2 PET for identifying either breast lesions or metastatic lesions were higher than those for ^18^F-FDG PET/CT (95.7% vs. 87.0%, and 100% vs. 57.1%) (63). The relative uptake values of glucose metabolism and angiogenesis imaging were also investigated. In newly diagnosed or recurrent breast cancer, all SUV_max_ for ^18^F-FPPRGD2 were lower than those for ^18^F-FDG, although the differences were not statistically significant (63). Again, the ^18^F-Alfatide II SUV_max_ was also lower than the ^18^F-FDG SUV_max_ (3.77 ± 1.78 vs. 7.37 ± 4.48) in breast cancer lesions in the study of Wu et al., and the difference was significant (p <0.05). However, in HER2 (–) and ER(+) breast cancer lesions, ^18^F-Alfatide II uptake was higher than ^18^F-FDG uptake (88). It is worthy noting that RGD PET may neglect the distant metastasis in liver; for example, Kumar et al. found that ^68^Ga-DOTA-RGD was not retained in liver metastases in 9 patients (16%) that were discovered with ^18^F-FDG imaging (119, 133). The high background activity of liver regions may contribute to this phenomenon (60).

The relative uptake values of RGD and FDG varied with the pathological or molecular phenotypes of breast cancer. Higher uptake values were observed in malignant compared with benign breast lesions on both ^18^F-Alfatide II and ^18^F-FDG imaging (all p <0.05). However, ^18^F-Alfatide II imaging showed a higher sensitivity (97.6% vs. 78.6%), lower specificity (54.5% vs. 81.8%), lower Youden index (52.1% vs. 60.4%), and lower area under the curve (0.738 vs. 0.838) on SUV_max_ analysis. These results indicate that ^18^F-Alfatide II has comparable diagnostic value to ^18^F-FDG, but may not be superior for identification of breast cancer (88). Concerning the molecular phenotypes in breast cancer patients, the SUV_max_ from ^68^Ga-NOTA-RGD-BBN (^68^Ga-BBN-RGD) PET was lower in ER(−) primary lesions than in ER(+) primary lesions (p <0.01). Additionally, ^18^F-Alfatide II uptake in the triple-negative subtype was significantly lower than that those in the luminal A and luminal B subtypes. However, no significant differences in uptake of either ^18^F-Alfatide II or ^68^Ga-BBN-RGD were observed between HER2(+) and HER2(−) subtypes in either patients with suspected breast cancer on screening mammography or patients who underwent breast cancer radical mastectomy (p = 0.6589) (88, 116). In patients with large or locally advanced IDC, the RGD SUV_max_ and SUV_mean_ differed significantly between the HER2(+) and HER2(−) groups (2.90 ± 0.75 vs. 2.42 ± 0.59, p = 0.04 for SUV_max_; 1.95 ± 0.53 vs. 1.60 ± 0.38, p = 0.04 for SUV_mean_) and showed significantly higher values in the ER/PR(+), HER2(−) and ER/PR(+), HER2(+) subgroups versus the ER/PR(−), HER2(−) subgroup in large or locally advanced IDC. ^18^F-FDG uptake was higher in HER2(+) breast cancer lesions than in the other 3 subtypes (triple-negative subtype, luminal A and luminal B subtypes) (88, 89). Both RGD-based tracers offer great performance for identifying breast cancer, but not superior to ^18^F-FDG. However, RGD-based tracers may be superior to ^18^F-FDG in detecting breast cancer that is strongly ER(+) and HER2(−). This implies that differing HER2 status, even if the hormonal receptor status is the same, can cause a difference in FDG uptake. At the same time, different hormonal receptor status can cause a difference in RGD uptake, even if the HER2 status is the same.

### Hepatocellular Carcinoma

As described above for the biodistribution of RGD-based tracers in the liver, two out of nine patients showed lower tracer accumulation in the HCC lesion as compared to the remaining liver parenchyma (72, 73). A similar lack of RGD-based tracer uptake was reported in liver metastases in breast cancer patients (60, 119). These findings were unexpected based on results demonstrating that 77% of investigated HCC specimens were highly vascularized with integrin αvβ3 expression compared with only 22% of normal liver tissue showing detectable αvβ3 expression (134), supporting the assumption that RGD uptake should be relatively higher in HCC than in normal liver tissue. The contradictory hypothesis that high RGD uptakes in HCC and later observations of hypointense tumors in the liver needs to be further clarified. The relatively clear background for ^18^F-FDG imaging in the liver appears to result from elevated glucose-6-phosphatase levels in liver cells. Overall, the research to date indicates the unsuitability and associated challenges of RGD-based imaging for HCC tumors or diseases with frequent liver metastases.

### Rectal Carcinoma

A study comparing ^18^F-FPRGD2 and ^18^F-FDG imaging in LARC patients reported that all primary LARC lesions with obvious ^18^F-FDG accumulation (SUV_max_, 16.5 ± 8) could also be detected by ^18^F-FPRGD2 uptake (SUV_max_, 5.4 ± 1.5), even though ^18^F-FPRGD2 uptake was lower than that of ^18^F-FDG (p <0.001). However, only 18 (51%) pelvic lymph nodes showed ^18^F-FPRGD2 uptake among 35 pelvic lymph nodes detected by ^18^F-FDG imaging. A moderate positive correlation was observed between ^18^F-FPRGD2 and FDG uptake (SUV_max_, Pearson’s r = 0.49, p = 0.0026; SUV_mean_, Pearson’s r = 0.54, p = 0.0007) (66).

### Renal Cancer

In a study of RGD-based imaging for renal cancer by Mena et al., all primary or metastatic renal tumors with a target tumor lesion larger than 2.0 cm could be detected by ^18^F-AH111585 PET/CT correctly, based on increased tracer retention with an average SUV_80%max_ of 6.4 ± 2.0, TBR of 1.5 ± 0.4 and TMR of 8.5 ± 3.4. ^18^F-AH111585 imaging also identified higher uptake values in chromophobe RCCs than in non-chromophobe RCCs (average SUV_80%max_, 8.2 ± 1.8 vs. 5.4 ± 1.4, p = 0.020; average TNR, 1.5 ± 0.4 vs. 0.9 ± 0.2, p = 0.029), and, similarly, αvβ3 integrin expression showed a strong correlation with the SUV_80%max_ in chromophobe and nonchromophobe RCC (61). In another study involving patients with metastatic RCC, the ^18^F-FPPRGD2 SUV_max_ was lower than that on ^18^F-FDG PET/CT (4.4 ± 2.9 vs. 7.8 ± 5.6, p <0.001), and the detection rates of metastatic RCC lesions were comparable between ^18^F-FPPRGD2 PET/CT (55/60) and ^18^F-FDG PET/CT (52/60) (97).

In the study by Withofs et al., ^18^F-FPRGD2 PET/CT allowed estimation of both intra- and interindividual variability of integrin αvβ3 expression successfully, the correlation between ^18^F-FPRGD2 uptake and αvβ3 expression varied among different cases. In whole tumor samples, ^18^F-FPRGD2 uptake correlated significantly with αvβ3 expression. In the subgroup analysis of ccRCC, the ^18^F-FPRGD2 PET signal correlated with integrin αvβ3 expression on tumor cells, whereas in the pRCC group, the signal correlated with integrin αvβ3 expression on blood vessels. Immunohistochemical examination on tumor cells showed that integrin αvβ3 expression was significantly higher on ccRCC than on pRCC (3.6 ± 2 vs. 2.14 ± 1.8, p = 0.0099). However, for ^18^F-FPRGD2 uptake as a reflection of integrin αvβ3 staining on entire tumors, the mean SUV_max_ did not different between ccRCC (4.1 ± 1.2) and pRCC (3.3 ± 0.7) (67). These results indicated that integrin αvβ3 may highly expressed on tumor cells in ccRCC and new blood vessels (endothelial cells) in pRCC. ^18^F-FPRGD2 PET/CT can reliably depict αvβ3 expression in renal tumors but is only representative of angiogenesis when tumor cells do not significantly express integrin αvβ3.

### Prostate Cancer

Neoplastic cells of human prostate cancer are characterized by the overexpression of both integrin αvβ3 and gastrin-releasing peptide receptor (GRPR) (135). ^68^Ga-BBN-RGD targets both αvβ3 and GRPR and thus was tested for its ability to improve the detection rate of primary prostate cancer. ^68^Ga-BBN-RGD PET/CT could successfully revealed 3 of 4 (75%) lesions and all 14 metastasized lymph nodes. It could also detect 20 bone lesions in seven patients, among which 11 showed a positive signal on ^99m^Tc-MDP bone scans and only 5 of these lesions were detectable with MRI (74). In a recently published clinical study in prostate cancer patients using ^18^F-Galacto-RGD PET, 58 of 74 (78.4%) bone lesions and 2 of 5 metastatic lymph nodes were identified. Thus, RGD imaging was feasible for achieving good visualization and detection of bone metastases due to low background activity in the surrounding normal bone tissue (136).

Importantly, prostate-specific membrane antigen (PSMA), a well-known target in prostate cancer, is not expressed in non-prostate tumors nor in healthy vasculature, but is expressed in the endothelial cells of tumor-associated neovasculature (137). PSMA expression appears to be prognostic biomarker in HNSCC, osteosarcoma, colorectal cancer, adenocarcinoma of the pancreas, lung cancer and others (138). In prostate cancer patients, the correlation of ^18^F-Galacto-RGD uptake and PSMA expression was also investigated. For all patients including one outlier or not with a very high PSMA value of 1,935 ng/ml, a significant and inverse correlation was identical between PSMA expression and ^18^F-Galacto-RGD SUV_mean_. Thus, RGD PET may serve as a new method for monitoring the status of PSMA.

### Cervical Cancer and Ovarian Cancer

In a study including 52 lesions in five patients with cervical cancer and ovarian cancer, ^18^F-FPPRGD2 imaging identified 48 lesions from 50 lesions on ^18^F-FDG imaging. Tracer uptake was significantly lower than ^18^F-FDG uptake in the lesions in terms of SUV_max_ (3.7 ± 1.3 vs. 6.0 ± 1.8, p <0.001), SUV_mean_ (2.6 ± 0.7 vs. 4.2 ± 1.3, p <0.001), TBR based on SUV_max_ (2.4 ± 1.0 vs. 2.6 ± 1.0, p <0.04), and TBR based on SUV_mean_ (1.9 ± 0.6 vs. 2.4 ± 1.0, p <0.003). Lesion accumulation of ^18^F-FPPRGD2 was mildly correlated with ^18^F-FDG uptake (SUV_max_ r = 0.39, p <0.005; SUV_mean_ r = 0.29, p <0.04) (90).

### Brain or Bone Metastasis

Almost all RGD-containing imaging agents showed no or very low tracer retention in normal brain tissue, as reported by preliminary studies and case reports, indicating that RGD-based radiotracer does not penetrate the BBB but can localize in tumor tissue through the disruption of the BBB (43, 61). In a study of RGD-based imaging of brain metastasis, ^18^F-Alfatide II PET could visualize all 20 brain lesions successfully, while only 10 lesions were visualized by ^18^F-FDG imaging, and 13 by CT. Similar to uptake at other tumor sites, ^18^F-FDG showed an overall higher SUV_max_ (10.0 ± 5.7) than ^18^F-Alfatide II (1.8 ± 1.1) in brain tumor lesions. However, ^18^F-Alfatide II showed a much better TNR (18.9 ± 14.1) than ^18^F-FDG (1.5 ± 0.5) in these brain lesions (47). A possible explanation is that the high physiologic glucose uptake in the surrounding normal brain tissues further enhanced RGD uptake, and low ^18^F-Alfatide II PET uptake in normal tissue may allow clear diagnosis and evaluation of tumor metastases in the brain (139). Another possible explanation for these results is that angiogenesis, the target of ^18^F-Alfatide II, is one of the key pathophysiologic processes in brain metastases (47, 140).

In a study of RGD-based imaging of bone metastasis in breast cancer patients, 18 lesions (33%) were detected with both ^68^Ga-DOTA-RGD and ^18^F-FDG imaging in (119). In another larger prospective study comparing ^18^F-Alfatide II and ^18^F-FDG PET, bone metastases were divided into four groups: osteolytic, osteoblastic, mixed and bone marrow based on their characteristics (141). In 11 patients with a total of 126 bone metastasis lesions, ^18^F-Alfatide II PET showed a higher positive detection rate than ^18^F-FDG PET (92% vs. 77%). In the subgroup analysis, ^18^F-Alfatide II PET was comparable to ^18^F-FDG PET/CT for the detection of osteolytic (100% vs. 90%) and mixed bone metastases (100% vs. 90%), but superior to ^18^F-FDG PET/CT for detecting osteoblastic (70% vs. 53%) and bone marrow lesions (98% vs. 77%). The sensitivity of ^18^F-Alfatide II PET/CT for osteolytic, mixed, and bone marrow lesions was nearly 100% (120). The higher positive detection rate of ^18^F-Alfatide II PET for bone marrow metastatic lesions may be due to either subclonal selection of integrin αvβ3-expressing tumor cell populations or upregulation of integrin αvβ3 in the bone microenvironment during the early phase of bone metastasis (142). Interestingly, RGD-containing tracer uptake was not lower than that of FDG in bone metastatic sites (4.27 ± 2.42 vs. 4.18 ± 2.58, p >0.05), which may be related to abundant integrin αvβ3 expression on the metastatic tumor cells, endothelial cells, and osteoclasts, whereas glycolysis is increased only within metastatic tumor cells and not in endothelial cells or osteoclasts (143, 144).

Both types of tracers showed significantly lower accumulation in osteoblastic lesions than that in osteolytic and mixed lesions (p <0.001 for all comparisons). The SUV_max_ of ^18^F-FDG in osteoblastic lesions was also significantly lower than that in bone marrow metastases (p <0.05). However, the ^18^F-Alfatide II SUV_max_ did not differ between osteoblastic lesions and bone marrow metastases. Another case report with ^68^Ga-DOTA-RGD2 imaging, it clearly depicted a left-sided frontal tumor without ^18^F-FDG PET/CT uptake. Considering the limitations of ^18^F-FDG PET in a setting of low glycolytic activity, ^68^Ga-DOTA-RGD2 PET/CT may be a clinically useful imaging modality for early detection of recurrent osteosarcoma (145).

## Definition of Tumor Subvolumes Using RGD-Containing Peptides as Radiotracers

For radiation treatment planning, imaging quality is vitally important for biological target volume definition. Beer et al. fused images from ^18^F-Galacto-RGD PET and MRI and/or multislice CT (MSCT) scanning in HNSCC patients. The mean gross tumor volume (GTV) was 26.4 ± 20.2 cm^3^, and when a SUV threshold >3.0 was applied, the mean tumor subvolume was only 3.4 ± 3.4 cm^3^, accounting for 13.1 ± 11.7% of the GTV (107). These results confirm the imaging quality and heterogeneous subvolume achieved with RGD-containing peptide tracers, which allows for adequate manual fusion with MRI or MSCT using anatomic landmarks. As displayed in a HNSCC patient population by Durante et al., ^68^Ga-NODAGA-RGD and ^18^F-FDG PET/CT individually led to inconsistent tumor subvolume delineation. The tracer avid tumor volume (TATV) calculated from RGD imaging tended to be larger than the metabolic tumor volume (MTV) calculated from ^18^F-FDG imaging when a 42% SUV_max_ fixed threshold similarly to MTV delineation was used (p = 0.085) (87). That difference in tumor delineation is assumed to be attributable to the difference in tracer targeting between FDG and RGD, as mostly homogeneous FDG uptake is significantly correlated with glucose metabolism and heterogeneous RGD uptake is associated with αvβ3 on angiogenesis. In addition, the physical properties of radionuclides fluorine-18 and gallium-68 also should be considered. At the same time, the 42% SUV_max_ of RGD was much lower than that of ^18^F-FDG, which may also have contributed to the calculation of a larger volume for the same tumor. In LARC cases examined with ^18^F-FPRGD2 imaging, the baseline integrin tumor volume (ITV) 70% (ITV_70%_, delineated using a threshold of 70% of the SUV_max_) was moderately correlated with the MTV_40%_ (delineated using a threshold of 40% of the SUV_max_) and total lesion glycolysis (calculated as SUV_mean_ × MTV_40%_) (66).

However, such results have not been reproduced in Lewis lung carcinoma (LLC) tumor-bearing C57BL/6 mice. The GTV determined from ^18^F-Alfatide PET was smaller and much closer to the pathological volume than that from ^18^F-FDG PET although a similar 40% of the SUV_max_ threshold was used (146). Several points are worth discussing regarding this difference. First, the expression pattern of αvβ3 varies among cancer types; it is mainly expressed on the neovasculature of HNSCC but on both the neovasculature and tumor cells in lung cancer (107, 111, 147). Secondly, different radionuclides were used in the two studies with ^18^F-Alfatide and ^68^Ga-NODAGA-RGD. Additionally, differences in metabolism between animals and humans may also influence the results. Importantly, it is unscientific to define the cutoff value for tumor subvolume delineation in reference to the established threshold for FDG. Instead, threshold optimization for estimating tumor volume based on a spatial comparison of αvβ3 expression on whole-tumor histological slices needs to be performed.

## Assessment of Therapeutic Response and Adverse Events Using RGD-Containing Peptides as Radiotracers

Because RGD is a marker of αvβ3 expression on angiogenesis which is of paramount importance for tumor oxygenation, RGD-containing PET tracers have been considered as a potentially valuable tool for monitoring treatment response and identifying patients less likely to respond to therapy. Several clinical pilot studies have explored their value in assessing treatment efficacy and adverse events, with encouraging results for both antiangiogenic therapy and chemoradiotherapy. Knowledge of the precision of repeated imaging parameters is a prerequisite for serial response measurements and, therefore, response to treatment. In the study by Sharma et al., solid tumor patients underwent two ^18^F-AH111585 imaging sessions within an interval of 10 days before commencement of therapy. No significant change in the SUV_peak_ was observed between the two scans, and the 95% reference range of spontaneous fluctuations was 35–39% (148). Measurements of ^18^F-FPPRGD2 uptake in normal organs also showed no significant changes between pre-, 1-week post- and 6-week post-therapy scans (149). Thus, with their protocol, the serial reproducibility of RGD-based tracer uptake supports further investigation of these novel tracers as biomarkers of treatment response (**Table 2**).

**Table 2:** Assessment of therapeutic response and adverse events using RGD-containing peptides as radiotracers.

Therapeutic regimen	Parameters	Prognosis	Threshold	Tracer agents	Tumor type	Reference
Single antiangiogenic therapy	higher baseline lesion SUV_mean_	better response and PFS	SUV_mean_ = 3.82	^18^F-Alfatide	locally advanced and metastatic malignancies	(150, 151)
	lower SUV_max_ in normal organs	less adverse events	SUV_max-liver_ = 4.57; SUV_max-spleen_ = 6.77; SUV_max-cardia_ = 2.10			
Pazopanib followed by combination therapy with paclitaxel	lower baseline lesion SUV_mean_	prolonged PFS	–	^18^F-AH111585	platinum-resistant/refractory ovarian cancer	(152)
	higher decreases of lesion SUV_mean_	better response	–			
Concurrent chemoradiotherapy	lower baseline and 3-week tumor SUV_max_ and TNR	better response	baseline SUV_max_ = 1.57; 3-week SUV_max_ = 1.35; 3-week TNR = 19.3	^18^F-Alfatide	GBM	(153)
Concurrent chemoradiotherapy	lower baseline tumor SUV_max_, SUV_peak_, TNR, TBR, TMR	better response	SUV_max_ = 5.65; SUV_peak_ = 4.46; TNR = 7.11; TBR = 5.41; TMR = 11.75	^18^F-Alfatide	advanced NSCLC	(154)
Concurrent chemoradiotherapy	lower 3-month tumor SUV_mean_	better response	–	^18^F-RDG-K5	locally advanced HNSCC	(99)
Concurrent long-course chemoradiotherapy	higher baseline SUV_max_ and SUV_mean_	better TRG	RGD SUV_max_ = 5.6; SUV_mean_ = 4.5 FDG SUV_max_ = 17; SUV_mean_ = 10.4	^18^F-FPRGD2 and ^18^F-FDG	locally advanced rectal cancer	(66)
	higher baseline SUV_max_	better PET response	–			

PFS, progression free survival; SUV_mean_, mean standard uptake values; SUV_max_, maximum standard uptake values; SUV_peak_, peak standard uptake values; TNR, tumor-to normal ratio; TBR, tumor-to-blood ratio; TMR, tumor-to-muscle ratio; TRG, tumour regression grade; GBM, glioblastoma multiform; NSCLC, non-small cell lung cancer; HNSCC, squamous cell carcinoma of head and neck.

Significant differences in RGD-based tracer uptake before and after antiangiogenic therapy have been reported in several studies (90, 97, 149). In GBM patients receiving bevacizumab-containing therapy, decreases of less than 15% in the SUV_max_ and angiogenic volumes from ^18^F-FPPRGD2 imaging at 1 week predicted recurrence and poor prognosis, while decreases of more than 50% predicted good outcomes to treatment (149). In another study including ovarian and cervical cancer, patients who experienced clinical disease progression, complete response, and partial response showed decreases in the lesional ^18^F-FPPRGD2 SUV_mean_ of 1.6, 25.2, and 7.9%, respectively, with similar decreases in the ^18^F-FDG SUV_mean_ of 9.4, 71.8, and 76.4%, respectively (90). The above studies mainly evaluated the changes in tracer uptake with the application of different antiangiogenic combination therapies, which may confound our understanding of RGD-based imaging for monitoring the response. Li et al. successfully investigated the relationships of ^18^F-Alfatide with the response to single antiangiogenic therapy in solid malignancies, higher pretreatment SUV_peak_ and SUV_mean_ were seen in responding tumors compared with non-responding tumors (4.98 ± 2.34 vs. 3.59 ± 1.44, p = 0.048; 3.71 ± 1.15 vs. 2.95 ± 0.49, p = 0.036). Cases with an ^18^F-Alfatide SUV_mean_ above 3.82 showed longer progression-free survival (PFS) than those with a SUV_mean_ less than 3.82. These results provided clinical evidence of the effectiveness of RGD-based PET at baseline for predicting the response to antiangiogenic therapy, suggesting this approach could potentially replace indicators based on change ratios in most previous studies (150). In addition, they also reported the predictive value of RGD-based imaging for antiangiogenic adverse events for the first time. Patients with low maximum ^18^F-Alfatide SUVs in the liver, spleen, and cardiac tissue were more likely to experience fatigue, hypertension, and nausea during Apatinib treatment (151). Thus, higher ^18^F-Alfatide uptake in specific normal organs predicted less adverse events, whereas higher uptake in tumor lesions predicted a better response to therapy. Higher accumulation in normal organs may indicate more regular, functional vasculature and sufficient blood flow, and higher accumulation within tumors may indicate greater expression of αvβ3 integrins (155, 156).

Results suggesting RGD-based tracer uptake at baseline can predict the response to antiangiogenic therapy have also been obtained in patients with platinum-resistant/refractory ovarian cancer (152). Of interest, a negative trend was observed between the baseline SUV_60,mean_ and PFS. There are several possible explanations for this contradictory observation. First, unlike the study by Li et al., the patients in this study received antiangiogenic combination therapy. It is well established that the addition of chemotherapy also enables a reduction in tumor size, which may account for the lack of association. Secondly, the PET response was assessed after 1 week, while the RECIST 1.1 response was evaluated after 3 months. Finally, preclinical work suggests that normalization of vessels occurs within hours and lasts 7–10 days, it is possible that performing the second PET scan at 7 days may have missed the neovascularization window (157). Overall, further research is needed to explore the potential of RGD-based tracers as biomarkers for response to antiangiogenic and antiangiogenic combination therapies.

The predictive value of RGD-based imaging for the response to chemoradiotherapy was also studied. Twenty-five newly diagnosed GBM patients who were ready for concurrent chemoradiotherapy (CCRT) 3–5 weeks after surgical resection were prospectively enrolled in a study by Zhang et al. The baseline ^18^F-Alfatide SUV_max_, the 3-week SUV_max_, and the TNR were found to be predictive of sensitivity to CCRT. The 3-week SUV_max_ (AUC = 0.846) was shown to be the best predictive parameter, with a 3-week SUV_max_ higher than 1.35 predicting worse efficacy (153). Further, Luan et al. explored the similar potential value of ^18^F-Alfatide PET/CT in locally advanced NSCLC. From their analysis, the pretreatment SUV_max_, SUV_peak_, TNR, TBR, and TMR were all significant parameters, with higher SUVs in non-responders than responders, and the TNR was shown to be an independent predictor (nonresponders vs. responders, 8.31 ± 0.61 vs. 6.53 ± 0.78, p <0.001) to CCRT (154). Chen et al. prospectively investigated the potential usefulness of RGD-K5 PET in HNSCC, patients whose primary tumors had lower accumulation of both tracers at 3 months achieved response to CCRT successfully (99). For LARC patients treated with CCRT, baseline ^18^F-FPRGD2 SUV_max_ and SUV_mean_ were negatively correlated with the tumor regression grade (TRG). The baseline SUV_max_ of both RGD and FDG imaging were significantly higher in TRG 0 cases than in the other patients. A cut-off value of 5.6 for RGD SUV_max_ identified TRG 0 status with 100% sensitivity and 66.7% specificity (AUC = 0.84) (66)..

From the research described above, it is interesting to note that RGD-based tracer uptake follows different trends according to differing treatment modalities. For antiangiogenic therapy, the higher uptake values may indicate a high density of effective target receptors and a better response to apatinib therapy. For CCRT, higher uptake values may represent high malignancy and severe hypoxia, which could contribute to increased chemoradiation resistance in tumors (158–160).

## Conclusion and Other Perspectives

A variety of radiolabeled, RGD-based PET imaging agents have been developed and extensively investigated in terms of their value for tumor detection and diagnosis, patient stratification, and monitoring of treatment response. According to promising results, these PET imaging peptides were transferred successfully from preclinical animal studies to clinical human trials. Different RGD-based PET imaging can be used in patients with glioma, lung cancer, esophageal cancer, breast cancer, renal carcinoma and other tumors to differentiate benign and malignant lesions and also pathological types and stages, and also can further distinguish molecular subtypes of breast cancer, delineate tumor volume, and predict the efficacy of radiochemotherapy and antiangiogenic therapy. The results regarding their diagnostic and predictive value are mostly derived from preliminary studies, and continued research is necessary to guarantee their development for clinical application.

In addition, RGD-based imaging was useful as a noninvasive surrogate parameter of cardiovascular disease. Normal angiogenesis under pathological conditions in cardiovascular diseases such as carotid atherosclerosis, myocardial infarction or reperfusion were also characterized by the overexpression of αvβ3 (3, 4, 161). In atherosclerotic lesions, highly expressed αvβ3 were found in both macrophages and activated endothelial cells (105, 162). The uptakes of ^68^Ga-DOTA-RGD and ^18^F-GalactoRGD PET increased in the atherosclerotic plaques in mice (95, 163). Clinically, RGD uptakes were heterogeneously distributed and significantly correlated with avβ3 integrin staining score in human atherosclerotic carotid plaques (164, 165). For ischemia tissue repair after myocardial infarction, αvβ3 is one of the necessary integrins involved in the formation of new capillaries (25, 166). RGD-based imaging of myocardial infarction observed consistent findings from studies of rats, specific RGD tracer accumulation uptake increased in vivo corresponding to infarcted and peri-infarct myocardial regions, and decreased after reperfusion (167–170). Tracer uptakes were expected to be used to monitor the process of myocardial repair (171–173). Makowski et al. confirmed the feasibility of ^18^F-Galacto-RGD to monitor vascular remodeling during evolving MI in human studies; tracer uptake was significantly correlated with infarct size and impairment of myocardial blood flow (174). Thus, RGD-based imaging of αvβ3 integrin expression could represent a novel promising imaging approach in determining the angiogenesis of carotid atherosclerosis and myocardial repair, which is potentially affecting the plaque vulnerability and left ventricular remodeling.

The RGD peptides also served as the new target for tumor treatment. RGD peptides could significantly reduce the functional vascular density, inhibit the adhesion and induce apoptosis of tumor cells according to inhibiting the interactions between integrins and their ligands (175–177). The RGD modified gene carriers and target drugs also showed special interest in delivery (178, 179). RGD peptides for imaging or tumor treatment all need to be further investigations for clinical translation.

It is important to note that contradictory reports about the correlation between RGD tracer uptake and αvβ3 expression level exist and that the ultimate meaning of αvβ3 expression in the context of angiogenesis is very complex and requires further characterization. The highly abnormal and dysfunctional vasculature created tumor hypoxic environment and impaired the ability of immune effector cells to penetrate solid tumors. In turn, stimulation of immune cell could also help normalize tumor vessels (180). Other studies have demonstrated the expression of integrin αvβ3 on activated macrophages by different methods (162, 181, 182). Therefore, RGD-based imaging targeting tumor neovascularization may offer a strategy to monitor the tumor microenvironment status and therapeutic response during immune checkpoint blockade or combination treatment. Moreover, the development of the clinically available applications of RGD-based tracers has mainly focus on radionuclide-labeled tracers, approaches to develop molecularly targeted nanoparticles for molecular imaging should be explored. Additionally, the simultaneous acquisition of functional parameters by MRI and PET may help compensate for the shortcomings of these different approaches. A thorough and complete assessment of different aspects of angiogenesis observed via multimodal imaging and biomomics characteristics should be implemented as part of the concept of personalized functional medicine.

## Author Contributions

SY proposed the conception and edited the manuscript. LL participated and drafted the manuscript. XC and JY participated and edited the manuscript. All authors listed have made a substantial, direct, and intellectual contribution to the work and approved it for publication.

## Funding

This study was supported in part by the National Natural Science Foundation of China (grant nos. NSFC81872475 and NSFC82073345) and the Jinan Clinical Medicine Science and Technology Innovation Plan (202019060) to SY, the Academic Promotion Program of Shandong First Medical University (grant No. 2019ZL002) to JY, the National University of Singapore Start-up Grant (NUHSRO/2020/133/Startup/08), the NUS School of Medicine Nanomedicine Translational Research Programme (NUHSRO/2021/034/TRP/09/Nanomedicine), and the NUS School of Medicine Kickstart Initiative (NUHSRO/2021/044/Kickstart/09/LOA) to XC.

## Conflict of Interest

The authors declare that the research was conducted in the absence of any commercial or financial relationships that could be construed as a potential conflict of interest.

## Publisher’s Note

All claims expressed in this article are solely those of the authors and do not necessarily represent those of their affiliated organizations, or those of the publisher, the editors and the reviewers. Any product that may be evaluated in this article, or claim that may be made by its manufacturer, is not guaranteed or endorsed by the publisher.

## References

[1] Auguste P Lemiere S Larrieu-Lahargue F Bikfalvi A. Molecular Mechanisms of Tumor Vascularization. Crit Rev Oncol/Hematol (2005) 54(1):53–61. doi: 10.1016/j.critrevonc.2004.11.006 15780907

[2] Risau W Flamme I. Vasculogenesis. Annu Rev Cell Dev Biol (1995) 11(1):73–91. doi: 10.1146/annurev.cb.11.110195.000445 8689573

[3] Carmeliet P Ng Y-S Nuyens D Theilmeier G Brusselmans K Cornelissen I. Impaired Myocardial Angiogenesis and Ischemic Cardiomyopathy in Mice Lacking the Vascular Endothelial Growth Factor Isoforms VEGF 164 and VEGF 188. Nat Med (1999) 5(5):495–502. doi: 10.1038/8379 10229225

[4] Ferrara N Alitalo K. Clinical Applications of Angiogenic Growth Factors and Their Inhibitors. Nat Med (1999) 5(12):1359–64. doi: 10.1038/70928 10581076

[5] Fiedler W Graeven U Ergün SL Verago S Kilic N Stockschläder M. Vascular Endothelial Growth Factor, a Possible Paracrine Growth Factor in Human Acute Myeloid Leukemia. Blood (1997) 89(6):1870–5. doi: 10.1182/blood.V89.6.1870 9058706

[6] Foss HD Araujo I Demel G Klotzbach H Hummel M Stein H. Expression of Vascular Endothelial Growth Factor in Lymphomas and Castleman’s Disease. J Pathol (1997) 183(1):44–50. doi: 10.1002/(SICI)1096-9896(199709)183:1<44::AID-PATH1103>3.0.CO;2-I 9370946

[7] Perez-Atayde AR Sallan SE Tedrow U Connors S Allred E Folkman J. Spectrum of Tumor Angiogenesis in the Bone Marrow of Children With Acute Lymphoblastic Leukemia. Am J Pathol (1997) 150(3):815. 9060819 PMC1857903

[8] Folkman J Merler E Abernathy C Williams G. Isolation of a Tumor Factor Responsible for Angiogenesis. J Exp Med (1971) 133(2):275. doi: 10.1084/jem.133.2.275 4332371 PMC2138906

[9] Soldi R Mitola S Strasly M Defilippi P Tarone G Bussolino F. Role of αvβ3 Integrin in the Activation of Vascular Endothelial Growth Factor Receptor-2. EMBO J (1999) 18(4):882–92. doi: 10.1093/emboj/18.4.882 PMC1171181 10022831

[10] Folkman J. Tumor Angiogenesis: Therapeutic Implications. N Engl J Med (1971) 285(21):1182–6. doi: 10.1056/NEJM197111182852108 4938153

[11] Holash J Wiegand S Yancopoulos G. New Model of Tumor Angiogenesis: Dynamic Balance Between Vessel Regression and Growth Mediated by Angiopoietins and VEGF. Oncogene (1999) 18(38):5356–62. doi: 10.1038/sj.onc.1203035 10498889

[12] Folkman J. Anti-Angiogenesis: New Concept for Therapy of Solid Tumors. Ann Surg (1972) 175(3):409. doi: 10.1097/00000658-197203000-00014 5077799 PMC1355186

[13] Gimbrone MA Jr. Leapman SB Cotran RS Folkman J. Tumor Dormancy *In Vivo* by Prevention of Neovascularization. J Exp Med (1972) 136(2):261–76. doi: 10.1084/jem.136.2.261 PMC2139203 5043412

[14] Folkman J. Angiogenesis in Cancer, Vascular, Rheumatoid and Other Disease. Nat Med (1995) 1(1):27–30. doi: 10.1038/nm0195-27 7584949

[15] Algire G Chalkley H. Vascular Reactions of Nod and Malignant Tissues *In Vivo.* 1. *Vascular Recations of Mice to Wounds and to Normal and Neoplastic Transplants* . J Natl Cancer Inst (1945) 6:73–85. doi: 10.1093/jnci/6.1.73

[16] Ruoslahti E. RGD and Other Recognition Sequences for Integrins. Annu Rev Cell Dev Biol (1996) 12(1):697–715. doi: 10.1146/annurev.cellbio.12.1.697 8970741

[17] Pierschbacher MD Ruoslahti E. Cell Attachment Activity of Fibronectin can be Duplicated by Small Synthetic Fragments of the Molecule. Nature (1984) 309(5963):30–3. doi: 10.1038/309030a0 6325925

[18] Plow EF Haas TA Zhang L Loftus J Smith JW. Ligand Binding to Integrins. J Biol Chem (2000) 275(29):21785–8. doi: 10.1074/jbc.R000003200 10801897

[19] Takada Y Ye X Simon S. The Integrins. Genome Biol (2007) 8(5):1–9. doi: 10.1186/gb-2007-8-5-215 PMC1929136 17543136

[20] Arnaout M Mahalingam B Xiong J-P. Integrin Structure, Allostery, and Bidirectional Signaling. Annu Rev Cell Dev Biol (2005) 21:381–410. doi: 10.1146/annurev.cellbio.21.090704.151217 16212500

[21] Brooks PC. Role of Integrins in Angiogenesis. Eur J Cancer (1996) 32(14):2423–9. doi: 10.1016/S0959-8049(96)00381-4 9059330

[22] Ruoslahti E FGG. Integrin Signaling. Science (1999) 285(5430):1028–32. doi: 10.1126/science.285.5430.1028 10446041

[23] Brooks PC Clark RA Cheresh DA. Requirement of Vascular Integrin Alpha V Beta 3 for Angiogenesis. Science (1994) 264(5158):569–71. doi: 10.1126/science.7512751 7512751

[24] Marmé D. The Impact of Anti-Angiogenic Agents on Cancer Therapy. J Cancer Res Clin Oncol (2003) 129(11):607–20. doi: 10.1007/s00432-003-0488-9 PMC12161926 13680378

[25] Horton MA. The αvβ3 Integrin “Vitronectin Receptor”. Int J Biochem Cell Biol (1997) 29(5):721–5. doi: 10.1016/S1357-2725(96)00155-0 9251239

[26] Kumar C. Integrin αvβ3 as a Therapeutic Target for Blocking Tumor-Induced Angiogenesis. Curr Drug Targets (2003) 4(2):123–31. doi: 10.2174/1389450033346830 12558065

[27] Fox SB Gasparini G Harris AL. Angiogenesis: Pathological, Prognostic, and Growth-Factor Pathways and Their Link to Trial Design and Anticancer Drugs. Lancet Oncol (2001) 2(5):278–89. doi: 10.1016/S1470-2045(00)00323-5 11905783

[28] Gasparini G Brooks PC Biganzoli E Vermeulen PB Bonoldi E Dirix LY. Vascular Integrin Alpha (V) Beta3: A New Prognostic Indicator in Breast Cancer. Clin Cancer Res (1998) 4(11):2625–34. 9829725

[29] Vonlaufen A Wiedle G Borisch B Birrer S Luder P Imhof BA. Integrin α V β 3 Expression in Colon Carcinoma Correlates With Survival. Modern Pathol (2001) 14(11):1126–32. doi: 10.1038/modpathol.3880447 11706074

[30] Xiong J-P Stehle T Diefenbach B Zhang R Dunker R Scott DL. Crystal Structure of the Extracellular Segment of Integrin αvβ3. Science (2001) 294(5541):339–45. doi: 10.1126/science.1064535 PMC2885948 11546839

[31] Xiong J-P Stehle T Zhang R Joachimiak A Frech M Goodman SL. Crystal Structure of the Extracellular Segment of Integrin αvβ3 in Complex With an Arg-Gly-Asp Ligand. Science (2002) 296(5565):151–5. doi: 10.1126/science.1069040 11884718

[32] Sutcliffe-Goulden JL O’Doherty MJ Marsden PK Hart IR Marshall JF Bansal SS. Rapid Solid Phase Synthesis and Biodistribution of 18 F-Labelled Linear Peptides. Eur J Nucl Med Mol Imaging (2002) 29(6):754–9. doi: 10.1007/s00259-001-0756-3 12029548

[33] Haubner R Gratias R Diefenbach B Goodman SL Jonczyk A Kessler H. Structural and Functional Aspects of RGD-Containing Cyclic Pentapeptides as Highly Potent and Selective Integrin αvβ3 Antagonists. J Am Chem Soc (1996) 118(32):7461–72. doi: 10.1021/ja9603721

[34] Verrier S Pallu S Bareille R Jonczyk A Meyer J Dard M. Function of Linear and Cyclic RGD-Containing Peptides in Osteoprogenitor Cells Adhesion Process. Biomaterials (2002) 23(2):585–96. doi: 10.1016/S0142-9612(01)00145-4 11761179

[35] Janssen M Oyen WJ Massuger LF Frielink C Dijkgraaf I Edwards DS. Comparison of a Monomeric and Dimeric Radiolabeled RGD-Peptide for Tumor Targeting. Cancer Biother Radiopharm (2002) 17(6):641–6. doi: 10.1089/108497802320970244 12537667

[36] Chen X Tohme M Park R Hou Y Bading JR Conti PS. Micro-PET Imaging of αvβ3-Integrin Expression With 18F-Labeled Dimeric RGD Peptide. Mol Imaging (2004) 3(2):15353500200404109. doi: 10.1162/15353500200404109 15296674

[37] Chen X Liu S Hou Y Tohme M Park R Bading JR. MicroPET Imaging of Breast Cancer αv-Integrin Expression With 64Cu-Labeled Dimeric RGD Peptides. Mol Imaging Biol (2004) 6(5):350–9. doi: 10.1016/j.mibio.2004.06.004 15380745

[38] Liu S. Radiolabeled Multimeric Cyclic RGD Peptides as Integrin αvβ3 Targeted Radiotracers for Tumor Imaging. Mol Pharm (2006) 3(5):472–87. doi: 10.1021/mp060049x 17009846

[39] Timpe JM. Effects of Adeno-Associated Virus on Adenovirus Replication and Cell Viability. USA: University of Toledo (2006). 10.1128/JVI.00198-06 PMC1563798 16873238

[40] Haubner R. α V β 3-Integrin Imaging: A New Approach to Characterise Angiogenesis? Eur J Nucl Med Mol Imaging (2006) 33(1):54–63. doi: 10.1007/s00259-006-0136-0 16791598

[41] Debordeaux F Chansel-Debordeaux L Pinaquy J-B Fernandez P Schulz J. What About αvβ3 Integrins in Molecular Imaging in Oncology? Nucl Med Biol (2018) 62:31–46. doi: 10.1016/j.nucmedbio.2018.04.006 29807242

[42] Zhou Y Chakraborty S Liu S. Radiolabeled Cyclic RGD Peptides as Radiotracers for Imaging Tumors and Thrombosis by SPECT. Theranostics (2011) 1:58–82. doi: 10.7150/thno/v01p0058 21547153 PMC3086616

[43] Doss M Kolb HC Zhang JJ Bélanger M-J Stubbs JB Stabin MG. Biodistribution and Radiation Dosimetry of the Integrin Marker 18F-RGD-K5 Determined From Whole-Body PET/CT in Monkeys and Humans. J Nucl Med (2012) 53(5):787–95. doi: 10.2967/jnumed.111.088955 PMC7607669 22499613

[44] Wu Z Li Z-B Cai W He L Chin FT Li F. 18 F-Labeled Mini-PEG Spacered RGD Dimer (18 F-FPRGD2): Synthesis and microPET Imaging of α V β 3 Integrin Expression. Eur J Nucl Med Mol Imaging (2007) 34(11):1823–31. doi: 10.1007/s00259-007-0427-0 PMC4167588 17492285

[45] Mittra ES Goris ML Iagaru AH Kardan A Burton L Berganos R. Pilot Pharmacokinetic and Dosimetric Studies of 18F-FPPRGD2: A PET Radiopharmaceutical Agent for Imaging αvβ3 Integrin Levels. Radiology (2011) 260(1):182–91. doi: 10.1148/radiol.11101139 PMC3121013 21502381

[46] Wan W Guo N Pan D Yu C Weng Y Luo S. First Experience of 18F-Alfatide in Lung Cancer Patients Using a New Lyophilized Kit for Rapid Radiofluorination. J Nucl Med (2013) 54(5):691–8. doi: 10.2967/jnumed.112.113563 PMC3683452 23554506

[47] Yu C Pan D Mi B Xu Y Lang L Niu G. 18 F-Alfatide II PET/CT in Healthy Human Volunteers and Patients With Brain Metastases. Eur J Nucl Med Mol Imaging (2015) 42(13):2021–8. doi: 10.1007/s00259-015-3118-2 PMC4626365 26121930

[48] Jeong JM Hong MK Chang YS Lee Y-S Kim YJ Cheon GJ. Preparation of a Promising Angiogenesis PET Imaging Agent: 68Ga-Labeled C (RGDyK)–isothiocyanatobenzyl-1, 4, 7-Triazacyclononane-1, 4, 7-Triacetic Acid and Feasibility Studies in Mice. J Nucl Med (2008) 49(5):830–6. doi: 10.2967/jnumed.107.047423 18413379

[49] Decristoforo C Hernandez Gonzalez I Carlsen J Rupprich M Huisman M Virgolini I. 68Ga- and 111In-Labelled DOTA-RGD Peptides for Imaging of Alphavbeta3 Integrin Expression. Eur J Nucl Med Mol Imaging (2008) 35(8):1507–15. doi: 10.1007/s00259-008-0757-6 18369617

[50] Knetsch PA Petrik M Griessinger CM Rangger C Fani M Kesenheimer C. [68ga]NODAGA-RGD for Imaging Alphavbeta3 Integrin Expression. Eur J Nucl Med Mol Imaging (2011) 38(7):1303–12. doi: 10.1007/s00259-011-1778-0 21487838

[51] Liu Z Niu G Wang F Chen X. (68)Ga-Labeled NOTA-RGD-BBN Peptide for Dual Integrin and GRPR-Targeted Tumor Imaging. Eur J Nucl Med Mol Imaging (2009) 36(9):1483–94. doi: 10.1007/s00259-009-1123-z 19360404

[52] Zheng Y Wang H Tan H Cui X Yao S Zang J. Evaluation of Lung Cancer and Neuroendocrine Neoplasm in a Single Scan by Targeting Both Somatostatin Receptor and Integrin αvβ3. Clin Nucl Med (2019) 44(9):687–94. doi: 10.1097/RLU.0000000000002680 31274560

[53] Dijkgraaf I Kruijtzer JA Liu S Soede AC Oyen WJ Corstens FH. Improved Targeting of the Alpha(V)Beta (3) Integrin by Multimerisation of RGD Peptides. Eur J Nucl Med Mol Imaging (2007) 34(2):267–73. doi: 10.1007/s00259-006-0180-9 16909226

[54] Lang L Li W Guo N Ma Y Zhu L Kiesewetter DO. Comparison Study of [18F] FAl-NOTA-PRGD2,[18F] FPPRGD2, and [68Ga] Ga-NOTA-PRGD2 for PET Imaging of U87MG Tumors in Mice. Bioconjugate Chem (2011) 22(12):2415–22. doi: 10.1021/bc200197h PMC3244506 22026940

[55] Oxboel J Brandt-Larsen M Schjoeth-Eskesen C Myschetzky R El-Ali HH Madsen J. Comparison of Two New Angiogenesis PET Tracers 68Ga-NODAGA-E[c(RGDyK)]2 and (64)Cu-NODAGA-E[c(RGDyK)]2; *In Vivo* Imaging Studies in Human Xenograft Tumors. Nucl Med Biol (2014) 41(3):259–67. doi: 10.1016/j.nucmedbio.2013.12.003 24417983

[56] Haubner R Weber WA Beer AJ Vabuliene E Reim D Sarbia M. Noninvasive Visualization of the Activated αvβ3 Integrin in Cancer Patients by Positron Emission Tomography and [18F] Galacto-RGD. PloS Med (2005) 2(3):e70. doi: 10.1371/journal.pmed.0020070 15783258 PMC1069665

[57] Beer AJ Haubner R Goebel M Luderschmidt S Spilker ME Wester H-J. Biodistribution and Pharmacokinetics of the αvβ3-Selective Tracer 18F-Galacto-RGD in Cancer Patients. J Nucl Med (2005) 46(8):1333–41. 16085591

[58] Beer AJ Haubner R Wolf I Goebel M Luderschmidt S Niemeyer M. PET-Based Human Dosimetry of 18F-Galacto-RGD, a New Radiotracer for Imaging αvβ3 Expression. J Nucl Med (2006) 47(5):763–9. 16644745

[59] McParland BJ Miller MP Spinks TJ Kenny LM Osman S Khela MK. The Biodistribution and Radiation Dosimetry of the Arg-Gly-Asp Peptide 18F-AH111585 in Healthy Volunteers. J Nucl Med (2008) 49(10):1664–7. doi: 10.2967/jnumed.108.052126 18794263

[60] Kenny LM Coombes RC Oulie I Contractor KB Miller M Spinks TJ. Phase I Trial of the Positron-Emitting Arg-Gly-Asp (RGD) Peptide Radioligand 18F-AH111585 in Breast Cancer Patients. J Nucl Med (2008) 49(6):879–86. doi: 10.2967/jnumed.107.049452 18483090

[61] Mena E Owenius R Turkbey B Sherry R Bratslavsky G Macholl S. [18 F] Fluciclatide in the *In Vivo* Evaluation of Human Melanoma and Renal Tumors Expressing α V β 3 and α V β 5 Integrins. Eur J Nucl Med Mol Imaging (2014) 41(10):1879–88. doi: 10.1007/s00259-014-2791-x PMC6336392 24973039

[62] Mammen M Choi SK Whitesides GM. Polyvalent Interactions in Biological Systems: Implications for Design and Use of Multivalent Ligands and Inhibitors. Angew Chem Int Ed (1998) 37(20):2754–94. doi: 10.1002/(SICI)1521-3773(19981102)37:20<2754::AID-ANIE2754>3.0.CO;2-3 29711117

[63] Iagaru A Mosci C Shen B Chin FT Mittra E Telli ML. 18f-FPPRGD2 PET/CT: Pilot Phase Evaluation of Breast Cancer Patients. Radiology (2014) 273(2):549–59. doi: 10.1148/radiol.14140028 25033190

[64] Liu S Liu Z Chen K Yan Y Watzlowik P Wester H-J. 18 F-Labeled Galacto and PEGylated RGD Dimers for PET Imaging of α V β 3 Integrin Expression. Mol Imaging Biol (2010) 12(5):530–8. doi: 10.1007/s11307-009-0284-2 PMC2999579 19949981

[65] Minamimoto R Jamali M Barkhodari A Mosci C Mittra E Shen B. Biodistribution of the 18 F-FPPRGD 2 PET Radiopharmaceutical in Cancer Patients: An Atlas of SUV Measurements. Eur J Nucl Med Mol Imaging (2015) 42(12):1850–8. doi: 10.1007/s00259-015-3096-4 26062933

[66] Withofs N Martinive P Vanderick J Bletard N Scagnol I Mievis F. [18 F] FPRGD 2 PET/CT Imaging of Integrin α V β 3 Levels in Patients With Locally Advanced Rectal Carcinoma. Eur J Nucl Med Mol Imaging (2016) 43(4):654–62. doi: 10.1007/s00259-015-3219-y 26490751

[67] Withofs N Signolle N Somja J Lovinfosse P Nzaramba EM Mievis F. 18f-FPRGD2 PET/CT Imaging of Integrin αvβ3 in Renal Carcinomas: Correlation With Histopathology. J Nucl Med (2015) 56(3):361–4. doi: 10.2967/jnumed.114.149021 25655629

[68] Zhang X Xiong Z Wu Y Cai W Tseng JR Gambhir SS. Quantitative PET Imaging of Tumor Integrin Alphavbeta3 Expression With 18F-FRGD2. J Nucl Med (2006) 47(1):113–21. PMC4160026 16391195

[69] Lang L Ma Y Kiesewetter DO Chen X. Stability Analysis of Glutamic Acid Linked Peptides Coupled to NOTA Through Different Chemical Linkages. Mol Pharm (2014) 11(11):3867–74. doi: 10.1021/mp400706q PMC4224566 24533430

[70] Guo N Lang L Li W Kiesewetter DO Gao H Niu G. Quantitative Analysis and Comparison Study of [18F] AlF-NOTA-PRGD2,[18F] FPPRGD2 and [68Ga] Ga-NOTA-PRGD2 Using a Reference Tissue Model. PloS One (2012) 7(5):e37506. doi: 10.1371/journal.pone.0037506 22624041 PMC3356326

[71] Ebenhan T Schoeman I Rossouw DD Grobler A Marjanovic-Painter B Wagener J. Evaluation of a Flexible NOTA-RGD Kit Solution Using Gallium-68 From Different 68 Ge/68 Ga-Generators: Pharmacokinetics and Biodistribution in Nonhuman Primates and Demonstration of Solitary Pulmonary Nodule Imaging in Humans. Mol Imaging Biol (2017) 19(3):469–82. doi: 10.1007/s11307-016-1014-1 27743211

[72] Haubner R Finkenstedt A Stegmayr A Rangger C Decristoforo C Zoller H. [68 Ga] NODAGA-RGD–Metabolic Stability, Biodistribution, and Dosimetry Data From Patients With Hepatocellular Carcinoma and Liver Cirrhosis. Eur J Nucl Med Mol Imaging (2016) 43(11):2005–13. doi: 10.1007/s00259-016-3396-3 PMC5007270 27164900

[73] Vatsa R Bhusari P Kumar S Chakraborty S Dash A Singh G. Integrin αvβ3 as a Promising Target to Image Neoangiogenesis Using in-House Generator-Produced Positron Emitter 68Ga-Labeled DOTA-Arginine-Glycine-Aspartic Acid (RGD) Ligand. Cancer Biother Radiopharm (2015) 30(5):217–24. doi: 10.1089/cbr.2014.1781 26083951

[74] Zhang J Niu G Lang L Li F Fan X Yan X. Clinical Translation of a Dual Integrin αvβ3–and Gastrin-Releasing Peptide Receptor–Targeting PET Radiotracer, 68Ga-BBN-RGD. J Nucl Med (2017) 58(2):228–34. doi: 10.2967/jnumed.116.177048 PMC5288740 27493267

[75] Li Z-B Chen K Chen X. 68 Ga-Labeled Multimeric RGD Peptides for microPET Imaging of Integrin α V β 3 Expression. Eur J Nucl Med Mol Imaging (2008) 35(6):1100–8. doi: 10.1007/s00259-007-0692-y 18204838

[76] Chakraborty S Chakravarty R Vatsa R Bhusari P Sarma H Shukla J. Toward Realization of ‘Mix-and-Use’Approach in 68Ga Radiopharmacy: Preparation, Evaluation and Preliminary Clinical Utilization of 68Ga-Labeled NODAGA-Coupled RGD Peptide Derivative. Nucl Med Biol (2016) 43(1):116–23. doi: 10.1016/j.nucmedbio.2015.09.010 26527030

[77] Lobeek D Rijpkema M Terry S Molkenboer-Kuenen JD Joosten L Van Genugten E. Imaging Angiogenesis in Patients With Head and Neck Squamous Cell Carcinomas by [68Ga] Ga-DOTA-E-[C (RGDfK)] 2 PET/Ct. Eur J Nucl Med Mol Imaging (2020) 47(11):2647. doi: 10.1007/s00259-020-04766-2 32198613 PMC7515959

[78] López-Rodríguez V Galindo-Sarco C García-Pérez FO Ferro-Flores G Arrieta O Ávila-Rodríguez MA. PET-Based Human Dosimetry of the Dimeric αvβ3 Integrin Ligand 68Ga-DOTA-E-[C (RGDfK)] 2, a Potential Tracer for Imaging Tumor Angiogenesis. J Nucl Med (2016) 57(3):404–9. doi: 10.2967/jnumed.115.161653 26585063

[79] Wu Y Zhang X Xiong Z Cheng Z Fisher DR Liu S. microPET Imaging of Glioma Integrin {Alpha}V{Beta}3 Expression Using (64)Cu-Labeled Tetrameric RGD Peptide. J Nucl Med (2005) 46(10):1707–18. 16204722

[80] Cunningham VJ Jones T. Spectral Analysis of Dynamic PET Studies. J Cereb Blood Flow Metab (1993) 13(1):15–23. doi: 10.1038/jcbfm.1993.5 8417003

[81] Cai W Sam Gambhir S Chen X. Multimodality Tumor Imaging Targeting Integrin αvβ3. Biotechniques (2005) 39(6):S14–25. doi: 10.2144/000112091 20158499

[82] Kuemmerle JF. Occupation of αvβ3-Integrin by Endogenous Ligands Modulates IGF-I Receptor Activation and Proliferation of Human Intestinal Smooth Muscle. Am J Physiol-Gastrointest Liver Physiol (2006) 290(6):G1194–202. doi: 10.1152/ajpgi.00345.2005 16195423

[83] Chin FT Shen B Liu S Berganos RA Chang E Mittra E. First Experience With Clinical-Grade [18 F] FPP (RGD) 2: An Automated Multi-Step Radiosynthesis for Clinical PET Studies. Mol Imaging Biol (2012) 14(1):88–95. doi: 10.1007/s11307-011-0477-3 21400112 PMC3617483

[84] Pedersen MW Holm S Lund EL Hojgaard L Kristjansen PE. Coregulation of Glucose Uptake and Vascular Endothelial Growth Factor (VEGF) in Two Small-Cell Lung Cancer (SCLC) Sublines *In Vivo* and *In Vitro* . Neoplasia (2001) 3(1):80–7. doi: 10.1038/sj.neo.7900133 PMC1505028 11326319

[85] Quartuccio N Treglia G Salsano M Mattoli MV Muoio B Piccardo A. The Role of Fluorine-18-Fluorodeoxyglucose Positron Emission Tomography in Staging and Restaging of Patients With Osteosarcoma. Radiol Oncol (2013) 47(2):97. doi: 10.2478/raon-2013-0017 23801904 PMC3691088

[86] Beer AJ Lorenzen S Metz S Herrmann K Watzlowik P Wester H-J. Comparison of Integrin αvβ3 Expression and Glucose Metabolism in Primary and Metastatic Lesions in Cancer Patients: A PET Study Using 18F-Galacto-RGD and 18F-FDG. J Nucl Med (2008) 49(1):22–9. doi: 10.2967/jnumed.107.045864 18077538

[87] Durante S Dunet V Gorostidi F Mitsakis P Schaefer N Delage J. Head and Neck Tumors Angiogenesis Imaging With 68 Ga-NODAGA-RGD in Comparison to 18 F-FDG PET/CT: A Pilot Study. EJNMMI Res (2020) 10(1):1–11. doi: 10.1186/s13550-020-00638-w 32382869 PMC7205972

[88] Wu J Wang S Zhang X Teng Z Wang J Yung BC. 18f-Alfatide II PET/CT for Identification of Breast Cancer: A Preliminary Clinical Study. J Nucl Med (2018) 59(12):1809–16. doi: 10.2967/jnumed.118.208637 PMC6910641 29700127

[89] Yoon H-J Kang KW Chun IK Cho N Im S-A Jeong S. Correlation of Breast Cancer Subtypes, Based on Estrogen Receptor, Progesterone Receptor, and HER2, With Functional Imaging Parameters From 68 Ga-RGD PET/CT and 18 F-FDG PET/Ct. Eur J Nucl Med Mol Imaging (2014) 41(8):1534–43. doi: 10.1007/s00259-014-2744-4 24652232

[90] Minamimoto R Karam A Jamali M Barkhodari A Gambhir SS Dorigo O. Pilot Prospective Evaluation of 18 F-FPPRGD 2 PET/CT in Patients With Cervical and Ovarian Cancer. Eur J Nucl Med Mol Imaging (2016) 43(6):1047–55. doi: 10.1007/s00259-015-3263-7 26611425

[91] Cheng W Wu Z Liang S Fu H Wu S Tang Y. Comparison of 18F-AIF-NOTA-PRGD2 and 18F-FDG Uptake in Lymph Node Metastasis of Differentiated Thyroid Cancer. PloS One (2014) 9(6):e100521. doi: 10.1371/journal.pone.0100521 24956393 PMC4067334

[92] Parihar AS Mittal BR Kumar R Shukla J Bhattacharya A. 68Ga-DOTA-RGD2 Positron Emission Tomography/Computed Tomography in Radioiodine Refractory Thyroid Cancer: Prospective Comparison of Diagnostic Accuracy With 18F-FDG Positron Emission Tomography/Computed Tomography and Evaluation Toward Potential Theranostics. Thyroid (2020) 30(4):557–67. doi: 10.1089/thy.2019.0450 31870227

[93] Vermeulen P Roland L Mertens V Van Marck E De Bruijn E Van Oosterom A. Correlation of Intratumoral Microvessel Density and P53 Protein Overexpression in Human Colorectal Adenocarcinoma. Microvasc Res (1996) 51(2):164–74. doi: 10.1006/mvre.1996.0018 8778572

[94] Kubota R Yamada S Kubota K Ishiwata K Tamahashi N Ido T. Intratumoral Distribution of Fluorine-18-Fluorodeoxyglucose *In Vivo*: High Accumulation in Macrophages and Granulation Tissues Studied by Microautoradiography. J Nucl Med (1992) 33(11):1972–80. 1432158

[95] Laitinen I Saraste A Weidl E Poethko T Weber AW Nekolla SG. Evaluation of αvβ3 Integrin-Targeted Positron Emission Tomography Tracer 18F-Galacto-RGD for Imaging of Vascular Inflammation in Atherosclerotic Mice. Circul: Cardiovasc Imaging (2009) 2(4):331–8. doi: 10.1161/CIRCIMAGING.108.846865 19808614

[96] Xuan S-H Zhou Y-G Pan J-Q Zhu W Xu P. Overexpression of Integrin αv in the Human Nasopharyngeal Carcinoma Associated With Metastasis and Progression. Cancer Biomark (2013) 13(5):323–8. doi: 10.3233/CBM-130361 PMC12928304 24440971

[97] Toriihara A Duan H Thompson HM Park S Hatami N Baratto L. 18 F-FPPRGD 2 PET/CT in Patients With Metastatic Renal Cell Cancer. Eur J Nucl Med Mol Imaging (2019) 46(7):1518–23. doi: 10.1007/s00259-019-04295-7 30850872

[98] Cho HJ Lee JD Park JY Yun M Kang WJ Walsh JC. First in Human Evaluation of a Newly Developed Integrin Binding PET Tracer, 18F-RGD-K5 in Patients With Breast Cancer: Comparison With 18F-FDG Uptake Pattern and Microvessel Density. Soc Nucl Med (2009).

[99] Chen S-H Wang H-M Lin C-Y Chang JT-C Hsieh C-H Liao C-T. RGD-K5 PET/CT in Patients With Advanced Head and Neck Cancer Treated With Concurrent Chemoradiotherapy: Results From a Pilot Study. Eur J Nucl Med Mol Imaging (2016) 43(9):1621–9. doi: 10.1007/s00259-016-3345-1 26922351

[100] Zheng K Liang N Zhang J Lang L Zhang W Li S. 68Ga-NOTA-PRGD2 PET/CT for Integrin Imaging in Patients With Lung Cancer. J Nucl Med (2015) 56(12):1823–7. doi: 10.2967/jnumed.115.160648 PMC5223097 26429958

[101] Paik J-Y Ko B-H Choe YS Choi Y Lee K-H Kim B-T. PMA-Enhanced Neutrophil [18F] FDG Uptake Is Independent of Integrin Occupancy But Requires PI3K Activity. Nucl Med Biol (2005) 32(6):561–6. doi: 10.1016/j.nucmedbio.2005.04.016 16026702

[102] Beer AJ Haubner R Sarbia M Goebel M Luderschmidt S Grosu AL. Positron Emission Tomography Using [18F] Galacto-RGD Identifies the Level of Integrin αvβ3 Expression in Man. Clin Cancer Res (2006) 12(13):3942–9. doi: 10.1158/1078-0432.CCR-06-0266 16818691

[103] Fidler IJ. Angiogenesis and Cancer Metastasis. Cancer J (Sudbury Mass) (2000) 6:S134–41. 10803828

[104] Eliceiri BP Cheresh DA. The Role of αv Integrins During Angiogenesis: Insights Into Potential Mechanisms of Action and Clinical Development. J Clin Invest (1999) 103(9):1227–30. doi: 10.1172/JCI6869 PMC408360 10225964

[105] Brooks PC Montgomery AM Rosenfeld M Reisfeld RA Hu T Klier G. Integrin αvβ3 Antagonists Promote Tumor Regression by Inducing Apoptosis of Angiogenic Blood Vessels. Cell (1994) 79(7):1157–64. doi: 10.1016/0092-8674(94)90007-8 7528107

[106] Wilder R. Integrin Alpha V Beta 3 as a Target for Treatment of Rheumatoid Arthritis and Related Rheumatic Diseases. Ann Rheum Dis (2002) 61(suppl 2):ii96–9. doi: 10.1136/ard.61.suppl_2.ii96 PMC1766704 12379637

[107] Beer AJ Grosu A-L Carlsen J Kolk A Sarbia M Stangier I. [18F] Galacto-RGD Positron Emission Tomography for Imaging of αvβ3 Expression on the Neovasculature in Patients With Squamous Cell Carcinoma of the Head and Neck. Clin Cancer Res (2007) 13(22):6610–6. doi: 10.1158/1078-0432.CCR-07-0528 18006761

[108] Beer AJ Niemeyer M Carlsen J Sarbia M Nährig J Watzlowik P. Patterns of αvβ3 Expression in Primary and Metastatic Human Breast Cancer as Shown by 18F-Galacto-RGD PET. J Nucl Med (2008) 49(2):255–9. doi: 10.2967/jnumed.107.045526 18199623

[109] Montet X Montet-Abou K Reynolds F Weissleder R Josephson L. Nanoparticle Imaging of Integrins on Tumor Cells. Neoplasia (2006) 8(3):214–22. doi: 10.1593/neo.05769 PMC1578521 16611415

[110] Felding-Habermann B Fransvea E O’Toole TE Manzuk L Faha B Hensler M. Involvement of Tumor Cell Integrin αvβ3 in Hematogenous Metastasis of Human Melanoma Cells. Clin Exp Metastasis (2002) 19(5):427–36. doi: 10.1023/A:1016377114119 12198771

[111] Kang F Wang Z Li G Wang S Liu D Zhang M. Inter-Heterogeneity and Intra-Heterogeneity of α V β 3 in Non-Small Cell Lung Cancer and Small Cell Lung Cancer Patients as Revealed by 68 Ga-RGD 2 PET Imaging. Eur J Nucl Med Mol Imaging (2017) 44(9):1520–8. doi: 10.1007/s00259-017-3696-2 28405726

[112] Zitzmann S Ehemann V Schwab M. Arginine-Glycine-Aspartic Acid (RGD)-Peptide Binds to Both Tumor and Tumor-Endothelial Cells *In Vivo* . Cancer Res (2002) 62(18):5139–43. 12234975

[113] Schnell O Krebs B Carlsen J Miederer I Goetz C Goldbrunner RH. Imaging of Integrin αvβ3 Expression in Patients With Malignant Glioma by [18F] Galacto-RGD Positron Emission Tomography. Neuro-oncology (2009) 11(6):861–70. doi: 10.1215/15228517-2009-024 PMC2802406 19401596

[114] Li D Zhao X Zhang L Li F Ji N Gao Z. 68Ga-PRGD2 PET/CT in the Evaluation of Glioma: A Prospective Study. Mol Pharm (2014) 11(11):3923–9. doi: 10.1021/mp5003224 PMC4224544 25093246

[115] Li D Zhang J Ji N Zhao X Zheng K Qiao Z. Combined 68ga-NOTA-PRGD2 and 18F-FDG PET/CT can Discriminate Uncommon Meningioma Mimicking High-Grade Glioma. Clin Nucl Med (2018) 43(9):648–54. doi: 10.1097/RLU.0000000000002233 30052597

[116] Zhang J Mao F Niu G Peng L Lang L Li F. 68Ga-BBN-RGD PET/CT for GRPR and Integrin αvβ3 Imaging in Patients With Breast Cancer. Theranostics (2018) 8(4):1121. doi: 10.7150/thno.22601 29464003 PMC5817114

[117] Du X Zhang Y Chen L Mi B You Q Xu Y. Comparing the Differential Diagnostic Values of 18F-Alfatide II PET/CT Between Tuberculosis and Lung Cancer Patients. Contrast Media Mol Imaging (2018) 2018:8194678. doi: 10.1155/2018/8194678 29670497 PMC5836463

[118] Dong Y Wei Y Chen G Huang Y Song P Liu S. Relationship Between Clinicopathological Characteristics and PET/CT Uptake in Esophageal Squamous Cell Carcinoma:[18 F] Alfatide Versus [18 F] FDG. Mol Imaging Biol (2019) 21(1):175–82. doi: 10.1007/s11307-018-1216-9 29869060

[119] Kumar S Rayamajhi S Shukla J Vatsa R Mittal B. Performance of Ga-68 DOTA RGD PET/CT for Detecting Lymph Nodal and Distant Metastasis in Breast Cancer: A Comparative Study With F-18 FDG PET/Ct. Soc Nucl Med (2015).

[120] Mi B Yu C Pan D Yang M Wan W Niu G. Pilot Prospective Evaluation of 18F-Alfatide II for Detection of Skeletal Metastases. Theranostics (2015) 5(10):1115. doi: 10.7150/thno.12938 26199649 PMC4508500

[121] Liu N Zhao W Hu X-D Gao S Yu Q Wang S. A Pilot Study on Imaging of Integrin αvβ3 With RGD PET/CT in Patients With Glioma. Soc Nucl Med (2015). doi: 10.1016/j.ijrobp.2015.07.787

[122] Schnell O Krebs B Wagner E Romagna A Beer AJ Grau SJ. Expression of Integrin αvβ3 in Gliomas Correlates With Tumor Grade and Is Not Restricted to Tumor Vasculature. Brain Pathol (2008) 18(3):378–86. doi: 10.1111/j.1750-3639.2008.00137.x PMC2607528 18394009

[123] Parihar AS Sood A Kumar R Bhusari P Shukla J Mittal BR. Novel Use of 177 Lu-DOTA-RGD 2 in Treatment of 68 Ga-DOTA-RGD 2-Avid Lesions in Papillary Thyroid Cancer With TENIS. Eur J Nucl Med Mol Imaging (2018) 45(10):1836–7. doi: 10.1007/s00259-018-4036-x 29713764

[124] Gao S Wu H Li W Zhao S Teng X Lu H. A Pilot Study Imaging Integrin αvβ3 With RGD PET/CT in Suspected Lung Cancer Patients. Eur J Nucl Med Mol Imaging (2015) 42(13):2029–37. doi: 10.1007/s00259-015-3119-1 26153145

[125] Zhou Y Gao S Huang Y Zheng J Dong Y Zhang B. A Pilot Study of 18 F-Alfatide PET/CT Imaging for Detecting Lymph Node Metastases in Patients With Non-Small Cell Lung Cancer. Sci Rep (2017) 7(1):1–7. doi: 10.1038/s41598-017-03296-6 28588317 PMC5460118

[126] Deppen S Putnam JB Jr Andrade G Speroff T Nesbitt JC Lambright ES. Accuracy of FDG-PET to Diagnose Lung Cancer in a Region of Endemic Granulomatous Disease. Ann Thorac Surg (2011) 92(2):428–33. doi: 10.1016/j.athoracsur.2011.02.052 PMC3186439 21592456

[127] Li L Zhao W Sun X Liu N Zhou Y Luan X. 18f-RGD PET/CT Imaging Reveals Characteristics of Angiogenesis in Non-Small Cell Lung Cancer. Trans Lung Cancer Res (2020) 9(4):1324. doi: 10.21037/tlcr-20-187 PMC7481644 32953507

[128] Van Der Gucht A Pomoni A Jreige M Allemann P Prior JO. 68Ga-NODAGA-RGDyK PET/CT Imaging in Esophageal Cancer: First-In-Human Imaging. Clin Nucl Med (2016) 41(11):e491–2. doi: 10.1097/RLU.0000000000001365 27607174

[129] Fox SB Leek RD Weekes MP Whitehouse RM Gatter KC Harris AL. Quantitation and Prognostic Value of Breast Cancer Angiogenesis: Comparison of Microvessel Density, Chalkley Count, and Computer Image Analysis. J Pathol (1995) 177(3):275–83. doi: 10.1002/path.1711770310 8551390

[130] Fox S Turner G Leek R Whitehouse R Gatter K Harris A. The Prognostic Value of Quantitative Angiogenesis in Breast Cancer and Role of Adhesion Molecule Expression in Tumor Endothelium. Breast Cancer Res Treat (1995) 36(2):219–26. doi: 10.1007/BF00666042 8534869

[131] Koukourakis MI Giatromanolaki A Sivridis E Fezoulidis I. Cancer Vascularization: Implications in Radiotherapy? Int J Radiat Oncol Biol Phys (2000) 48(2):545–53. doi: 10.1016/S0360-3016(00)00677-5 10974475

[132] Weidner N Folkman J Pozza F Bevilacqua P Allred EN Moore DH. Tumor Angiogenesis: A New Significant and Independent Prognostic Indicator in Early-Stage Breast Carcinoma. J Natl Cancer Inst (1992) 84(24):1875–87. doi: 10.1093/jnci/84.24.1875 1281237

[133] Vatsa R Ashwathanarayana AG Singh G Kavanal AJ Kumar S Rana N. A Comparison of Angiogenesis and Glycolytic Imaging in Patients With Clinical Suspected Locally Advanced Breast Cancer. Clin Nucl Med (2019) 44(8):e479–83. doi: 10.1097/RLU.0000000000002647 31274628

[134] Jin Y Chen J-n Feng Z-Y Zhang Z-g Fan W-z Wang Y. OPN and αvβ3 Expression Are Predictors of Disease Severity and Worse Prognosis in Hepatocellular Carcinoma. PloS One (2014) 9(2):e87930. doi: 10.1371/journal.pone.0087930 24498405 PMC3912195

[135] Beer M Montani M Gerhardt J Wild PJ Hany TF Hermanns T. Profiling Gastrin-Releasing Peptide Receptor in Prostate Tissues: Clinical Implications and Molecular Correlates. Prostate (2012) 72(3):318–25. doi: 10.1002/pros.21434 21739464

[136] Beer AJ Schwarzenböck SM Zantl N Souvatzoglou M Maurer T Watzlowik P. Non-Invasive Assessment of Inter-and Intrapatient Variability of Integrin Expression in Metastasized Prostate Cancer by PET. Oncotarget (2016) 7(19):28151. doi: 10.18632/oncotarget.8611 27058620 PMC5053716

[137] Bostwick DG Pacelli A Blute M Roche P Murphy GP. Prostate Specific Membrane Antigen Expression in Prostatic Intraepithelial Neoplasia and Adenocarcinoma: A Study of 184 Cases. Cancer (1998) 82(11):2256–61. doi: 10.1002/(SICI)1097-0142(19980601)82:11<2256::AID-CNCR22>3.0.CO;2-S 9610707

[138] Backhaus P Noto B Avramovic N Grubert LS Huss S Boegemann M. Targeting PSMA by Radioligands in Non-Prostate Disease—Current Status and Future Perspectives. Eur J Nucl Med Mol Imaging (2018) 45(5):860–77. doi: 10.1007/s00259-017-3922-y 29335762

[139] Peet AC Arvanitis TN Leach MO Waldman AD. Functional Imaging in Adult and Paediatric Brain Tumours. Nat Rev Clin Oncol (2012) 9(12):700–11. doi: 10.1038/nrclinonc.2012.187 23149894

[140] Zadeh G Guha A. Molecular Regulators of Angiogenesis in the Developing Nervous System and Adult Brain Tumors. Int J Oncol (2003) 23(3):557–65. doi: 10.3892/ijo.23.3.557 12888888

[141] Guise TA Mohammad KS Clines G Stebbins EG Wong DH Higgins LS. Basic Mechanisms Responsible for Osteolytic and Osteoblastic Bone Metastases. Clin Cancer Res (2006) 12(20):6213s–6s. doi: 10.1158/1078-0432.CCR-06-1007 17062703

[142] Liapis H Flath A Kitazawa S. Integrin Alpha V Beta 3 Expression by Bone-Residing Breast Cancer Metastases. Diagn Mol Pathol (1996) 5(2):127–35. doi: 10.1097/00019606-199606000-00008 8727100

[143] Suva LJ Washam C Nicholas RW Griffin RJ. Bone Metastasis: Mechanisms and Therapeutic Opportunities. Nat Rev Endocrinol (2011) 7(4):208–18. doi: 10.1038/nrendo.2010.227 PMC3134309 21200394

[144] Glaspy J Hawkins R Hoh C Phelps M. Use of Positron Emission Tomography in Oncology. Oncol (Williston Park NY) (1993) 7(7):41–6. 8347460

[145] Orunmuyi A Modiselle M Lengana T Ebenhan T Vorster M Sathekge M. 68 Gallium-Arginine-Glycine-Aspartic Acid and 18 F-Fluorodeoxyglucose Positron Emission Tomography/Computed Tomography in Chondroblastic Osteosarcoma of the Skull. Nucl Med Mol Imaging (2017) 51(3):271–3. doi: 10.1007/s13139-016-0400-6 PMC5567611 28878856

[146] Wei Y-C Hu X Gao Y Fu Z Zhao W Yu Q. Noninvasive Evaluation of Metabolic Tumor Volume in Lewis Lung Carcinoma Tumor-Bearing C57BL/6 Mice With Micro-PET and the Radiotracers 18F-Alfatide and 18F-FDG: A Comparative Analysis. PloS One (2015) 10(9):e0136195. doi: 10.1371/journal.pone.0136195 26352404 PMC4564167

[147] Terry SY Abiraj K Frielink C Van Dijk LK Bussink J Oyen WJ. Imaging Integrin αvβ3 on Blood Vessels With 111In-RGD2 in Head and Neck Tumor Xenografts. J Nucl Med (2014) 55(2):281–6. doi: 10.2967/jnumed.113.129668 24408894

[148] Sharma R Kallur KG Ryu JS Parameswaran RV Lindman H Avril N. Multicenter Reproducibility of 18F-Fluciclatide PET Imaging in Subjects With Solid Tumors. J Nucl Med (2015) 56(12):1855–61. doi: 10.2967/jnumed.115.158253 26383153

[149] Iagaru A Mosci C Mittra E Zaharchuk G Fischbein N Harsh G. Glioblastoma Multiforme Recurrence: An Exploratory Study of 18F FPPRGD2 PET/Ct. Radiology (2015) 277(2):497–506. doi: 10.1148/radiol.2015141550 25965900

[150] Li L Ma L Shang D Liu Z Yu Q Wang S. Pretreatment PET/CT Imaging of Angiogenesis Based on 18 F-RGD Tracer Uptake may Predict Antiangiogenic Response. Eur J Nucl Med Mol Imaging (2019) 46(4):940–7. doi: 10.1007/s00259-018-4143-8 30187104

[151] Li L Zheng J Liu Z Huang Y Xiao J Wang S. Pre-treatment 18F-RGD Uptake may Predict Adverse Events during Apatinib Antiangiogenic Therapy. Clin Oncol (2022) 18:S0936-6555(22)00017-6. doi: 10.1016/j.clon.2022.01.002 35063328

[152] Sharma R Valls PO Inglese M Dubash SR Chen M Gabra H. [18f] Fluciclatide PET as a Biomarker of Clinical Response to Combination Therapy of Pazopanib and Paclitaxel in Patients With Platinum-Resistant or Platinum-Refractory Advanced Ovarian Cancer: Results of a Phase Ib Study. Am Soc Clin Oncol (2019). doi: 10.1200/JCO.2019.37.15_suppl.3070

[153] Zhang H Liu N Gao S Hu X Zhao W Tao R. Can an 18F-ALF-NOTA-PRGD2 PET/CT Scan Predict Treatment Sensitivity to Concurrent Chemoradiotherapy in Patients With Newly Diagnosed Glioblastoma? J Nucl Med (2016) 57(4):524–9. doi: 10.2967/jnumed.115.165514 26514171

[154] Luan X Huang Y Gao S Sun X Wang S Ma L. 18 F-Alfatide PET/CT may Predict Short-Term Outcome of Concurrent Chemoradiotherapy in Patients With Advanced Non-Small Cell Lung Cancer. Eur J Nucl Med Mol Imaging (2016) 43(13):2336–42. doi: 10.1007/s00259-016-3505-3 PMC5095164 27631310

[155] Vaupel P Kallinowski F Okunieff P. Blood Flow, Oxygen Consumption and Tissue Oxygenation of Human Tumors. Oxygen Transport to Tissue XII (1990) 277:895–905. doi: 10.1007/978-1-4684-8181-5_103 2096691

[156] Brown JM Giaccia AJ. The Unique Physiology of Solid Tumors: Opportunities (and Problems) for Cancer Therapy. Cancer Res (1998) 58(7):1408–16. 9537241

[157] Jain RK. Normalizing Tumor Vasculature With Anti-Angiogenic Therapy: A New Paradigm for Combination Therapy. Nat Med (2001) 7(9):987–9. doi: 10.1038/nm0901-987 11533692

[158] Karar J Maity A. Modulating the Tumor Microenvironment to Increase Radiation Responsiveness. Cancer Biol Ther (2009) 8(21):1994–2001. doi: 10.4161/cbt.8.21.9988 19823031 PMC3965173

[159] Toustrup K Sørensen BS Nordsmark M Busk M Wiuf C Alsner J. Development of a Hypoxia Gene Expression Classifier With Predictive Impact for Hypoxic Modification of Radiotherapy in Head and Neck Cancer. Cancer Res (2011) 71(17):5923–31. doi: 10.1158/0008-5472.CAN-11-1182 21846821

[160] Niu G Chen X. RGD PET: From Lesion Detection to Therapy Response Monitoring. J Nucl Med (2016) 57(4):501–2. doi: 10.2967/jnumed.115.168278 PMC5241674 26609181

[161] Hoshiga M Alpers CE Smith LL Giachelli CM Schwartz SM. Alpha-V Beta-3 Integrin Expression in Normal and Atherosclerotic Artery. Circ Res (1995) 77(6):1129–35. doi: 10.1161/01.res.77.6.1129 7586225

[162] Antonov AS Kolodgie FD Munn DH Gerrity RG. Regulation of Macrophage Foam Cell Formation by αvβ3 Integrin: Potential Role in Human Atherosclerosis. Am J Pathol (2004) 165(1):247–58. doi: 10.1016/S0002-9440(10)63293-2 PMC1618536 15215180

[163] Johanna H Iina L Pauliina L Peter I Ian W Hege K. 68Ga-DOTA-RGD Peptide: Biodistribution and Binding Into Atherosclerotic Plaques in Mice. Eur J Nucl Med Mol Imaging (2009) 36(12):2058–67. doi: 10.1007/s00259-009-1220-z 19629477

[164] Golestani R Mirfeizi L Zeebregts CJ Westra J de Haas HJ Glaudemans AW. Feasibility of [18F]-RGD for *Ex Vivo* Imaging of Atherosclerosis in Detection of Alphavbeta3 Integrin Expression. J Nucl Cardiol (2015) 22(6):1179–86. doi: 10.1007/s12350-014-0061-8 25698472

[165] Beer AJ Pelisek J Heider P Saraste A Reeps C Metz S. PET/CT Imaging of Integrin Alphavbeta3 Expression in Human Carotid Atherosclerosis. JACC Cardiovasc Imaging (2014) 7(2):178–87. doi: 10.1016/j.jcmg.2013.12.003 24412187

[166] Arnaout MA Goodman SL Xiong JP. Coming to Grips With Integrin Binding to Ligands. Curr Opin Cell Biol (2002) 14(5):641–51. doi: 10.1016/s0955-0674(02)00371-x 12231361

[167] Higuchi T Bengel FM Seidl S Watzlowik P Kessler H Hegenloh R. Assessment of Alphavbeta3 Integrin Expression After Myocardial Infarction by Positron Emission Tomography. Cardiovasc Res (2008) 78(2):395–403. doi: 10.1093/cvr/cvn033 18256073

[168] Sherif HM Saraste A Nekolla SG Weidl E Reder S Tapfer A. Molecular Imaging of Early Alphavbeta3 Integrin Expression Predicts Long-Term Left-Ventricle Remodeling After Myocardial Infarction in Rats. J Nucl Med (2012) 53(2):318–23. doi: 10.2967/jnumed.111.091652 22302965

[169] Gao H Lang L Guo N Cao F Quan Q Hu S. PET Imaging of Angiogenesis After Myocardial Infarction/Reperfusion Using a One-Step Labeled Integrin-Targeted Tracer 18F-AlF-NOTA-Prgd2. Eur J Nucl Med Mol Imaging (2012) 39(4):683–92. doi: 10.1007/s00259-011-2052-1 PMC3319105 22274731

[170] Eo JS Paeng JC Lee S Lee YS Jeong JM Kang KW. Angiogenesis Imaging in Myocardial Infarction Using 68Ga-NOTA-RGD PET: Characterization and Application to Therapeutic Efficacy Monitoring in Rats. Coron Artery Dis (2013) 24(4):303–11. doi: 10.1097/MCA.0b013e3283608c32 23542160

[171] Menichetti L Kusmic C Panetta D Arosio D Petroni D Matteucci M. MicroPET/CT Imaging of Alphavbeta(3) Integrin *via* a Novel (6)(8)Ga-NOTA-RGD Peptidomimetic Conjugate in Rat Myocardial Infarction. Eur J Nucl Med Mol Imaging (2013) 40(8):1265–74. doi: 10.1007/s00259-013-2432-9 23674206

[172] Cai M Ren L Yin X Guo Z Li Y He T. PET Monitoring Angiogenesis of Infarcted Myocardium After Treatment With Vascular Endothelial Growth Factor and Bone Marrow Mesenchymal Stem Cells. Amino Acids (2016) 48(3):811–20. doi: 10.1007/s00726-015-2129-4 26592497

[173] Lang CI Doring P Gabel R Vasudevan P Lemcke H Muller P. [(68)Ga]-NODAGA-RGD Positron Emission Tomography (PET) for Assessment of Post Myocardial Infarction Angiogenesis as a Predictor for Left Ventricular Remodeling in Mice After Cardiac Stem Cell Therapy. Cells (2020) 9(6):1358. doi: 10.3390/cells9061358 PMC7349714 32486211

[174] Makowski MR Rischpler C Ebersberger U Keithahn A Kasel M Hoffmann E. Multiparametric PET and MRI of Myocardial Damage After Myocardial Infarction: Correlation of Integrin Alphavbeta3 Expression and Myocardial Blood Flow. Eur J Nucl Med Mol Imaging (2021) 48(4):1070–80. doi: 10.1007/s00259-020-05034-z PMC8041712 32970218

[175] Eliceiri BP Cheresh DA. The Role of Alphav Integrins During Angiogenesis: Insights Into Potential Mechanisms of Action and Clinical Development. J Clin Invest (1999) 103(9):1227–30. doi: 10.1172/JCI6869 PMC408360 10225964

[176] Chen Y Xu X Hong S Chen J Liu N Underhill CB. RGD-Tachyplesin Inhibits Tumor Growth. Cancer Res (2001) 61(6):2434–8. 11289111

[177] Mitjans F Meyer T Fittschen C Goodman S Jonczyk A Marshall JF. *In Vivo* Therapy of Malignant Melanoma by Means of Antagonists of Alphav Integrins. Int J Cancer (2000) 87(5):716–23. doi: 10.1002/1097-0215(20000901)87:5<716::AID-IJC14>3.0.CO;2-R 10925366

[178] Wang Z Lee TY Ho PC. A Novel Dextran-Oleate-Crgdfk Conjugate for Self-Assembly of Nanodrug. Nanomedicine (2012) 8(2):194–203. doi: 10.1016/j.nano.2011.06.006 21704594

[179] Vachutinsky Y Oba M Miyata K Hiki S Kano MR Nishiyama N. Antiangiogenic Gene Therapy of Experimental Pancreatic Tumor by Sflt-1 Plasmid DNA Carried by RGD-Modified Crosslinked Polyplex Micelles. J Control Rel (2011) 149(1):51–7. doi: 10.1016/j.jconrel.2010.02.002 20138936

[180] Huang Y Kim BY Chan CK Hahn SM Weissman IL Jiang W. Improving Immune–Vascular Crosstalk for Cancer Immunotherapy. Nat Rev Immunol (2018) 18(3):195–203. doi: 10.1038/nri.2017.145 29332937 PMC5922422

[181] Waldeck J Häger F Höltke C Lanckohr C von Wallbrunn A Torsello G. Fluorescence Reflectance Imaging of Macrophage-Rich Atherosclerotic Plaques Using an αvβ3 Integrin–Targeted Fluorochrome. J Nucl Med (2008) 49(11):1845–51. doi: 10.2967/jnumed.108.052514 18927332

[182] Yao Y Jiang Y Sheng Z Zhang Y An Y Yan F. Analysis of *in Situ* and *Ex Vivo* αvβ3 Integrin Expression During Experimental Carotid Atherogenesis. Int J Nanomed (2012) 7:641. doi: 10.2147/IJN.S28065 PMC3278228 22334786

