# Supplementary material for: Preliminary Clinical Application of RGD-Containing Peptides as PET Radiotracers for Imaging Tumors
Source: Front Oncol. 2022 Mar 2;12:837952. doi: 10.3389/fonc.2022.837952 (PMC8924613; doi:10.3389/fonc.2022.837952)
Supplement: Supplementary file 1 [file Table_1.docx]

| **Supplementary Table 1. Preliminarily clinically studied monomeric and multimeric RGD-based PET tracers** | | | | | |
| --- | --- | --- | --- | --- | --- |
| **Tracer name** | **No. of patients** | **Tumor types** | **Purpose** | **Year** | **Reference** |
| ^18^F-Galacto-RGD | 9 | malignant melanoma (n = 5), chondrosarcoma (n = 1), soft tissue sarcoma (n = 1), osseous metastasis of renal cell carcinoma (n = 1), villonodular synovitis of the knee (n = 1) | biodistribution, diagnosis | 2005 | ^42^ |
| ^18^F-Galacto-RGD | 19 | metastases of malignant melanoma (n = 7), sarcomas (n = 10), or osseous metastases (n = 2) | biodistribution, diagnosis | 2005 | ^43^ |
| ^18^F-Galacto-RGD | 18 | musculoskeletal tumors (n = 10), melanoma (n = 5), breast cancer (n = 2), head and neck cancer (n = 1) | safety, biodistribution | 2006 | ^44^ |
| ^18^F-Galacto-RGD | 19 | musculoskeletal system (n = 10), melanoma (n = 4), head and neck cancer (n = 2), glioblastoma (n = 2), breast cancer (n = 1) | diagnosis | 2006 | ^95^ |
| ^18^F-Galacto-RGD | 11 | squamous cell carcinoma of head and neck | diagnosis, definition of tumor subvolume | 2007 | ^100^ |
| ^18^F-Galacto-RGD | 18 | non-small cell lung cancer (n = 10), renal cell carcinoma (n = 2), rectal cancer (n = 2), others (n = 4) | diagnosis | 2008 | ^78^ |
| ^18^F-Galacto-RGD | 16 | primary (n = 12), metastasized breast cancer (n = 4) | diagnosis | 2008 | ^101^ |
| ^18^F-Galacto-RGD | 12 | primary or known recurrent glioblastoma | diagnosis | 2009 | ^106^ |
| ^18^F-Galacto-RGD | 12 | metastasized prostate cancer | diagnosis | 2016 | ^128^ |
| ^18^F-AH111585 | 8 | healthy volunteers | safety, biodistribution | 2008 | ^45^ |
| ^18^F-AH111585 | 7 | breast cancer | safety, diagnosis | 2008 | ^46^ |
| ^18^F-AH111585 | 18 | metastatic melanoma (n = 6), oncocytoma (n = 1), renal cell cancer (n = 11) | biodistribution, diagnosis | 2014 | ^47^ |
| ^18^F-AH111585 | 26 | 39 measurable lesions | reproducibility | 2015 | ^142^ |
| ^18^F-AH111585 | 14 | platinum-resistant/refractory ovarian cancer | therapeutic response | 2020 | ^148^ |
| ^18^F-RGD-K5 | 12 | breast cancer | diagnosis | 2009 | ^91^ |
| ^18^F-RGD-K5 | 4 | healthy volunteers | safety, biodistribution | 2012 | ^48^ |
| ^18^F-RGD-K5 | 9 | locally advanced head and neck cancer | diagnosis, therapeutic response | 2016 | ^92^ |
| ^18^F-FPRGD2 | 27 | renal carcinoma | diagnosis and differential diagnosis | 2015 | ^56^ |
| ^18^F-FPRGD2 | 32 | locally advanced rectal cancer | diagnosis, therapeutic response | 2016 | ^55^ |
| ^18^F-FPPRGD2 | 5 | healthy volunteers | biodistribution | 2011 | ^50^ |
| ^18^F-FPPRGD2 | 1 | healthy volunteer | safety, biodistribution | 2012 | ^75^ |
| ^18^F-FPPRGD2 | 8 | newly diagnosed or recurrent breast cancer | safety, diagnosis | 2014 | ^51^ |
| ^18^F-FPPRGD2 | 35 | cancer patients | biodistribution, diagnosis | 2015 | ^53^ |
| ^18^F-FPPRGD2 | 17 | glioblastoma multiforme | safety, diagnosis, therapeutic response | 2015 | ^143^ |
| ^18^F-FPPRGD2 | 6 | cervical cancer and ovarian cancer | biodistribution, diagnosis, therapeutic response | 2016 | ^82^ |
| ^18^F-FPPRGD2 | 7 | metastatic renal cell cancer | diagnosis and therapeutic response | 2019 | ^90^ |
| ^18^F-Alfatide | 9 | lung cancer | biodistribution, diagnosis | 2013 | ^58^ |
| ^18^F-Alfatide | 20 | differentiated thyroid cancer | diagnosis and differential diagnosis | 2014 | ^83^ |
| ^18^F-Alfatide | 13 | glioma | diagnosis and differential diagnosis | 2015 | ^110^ |
| ^18^F-Alfatide | 26 | suspected lung cancer | safety, biodistribution, diagnosis and differential diagnosis | 2015 | ^113^ |
| ^18^F-Alfatide | 25 | glioblastoma multiforme | diagnosis, therapeutic response | 2016 | ^150^ |
| ^18^F-Alfatide | 18 | non-small cell lung cancer | diagnosis, therapeutic response | 2016 | ^151^ |
| ^18^F-Alfatide | 13 | non-small cell lung cancer | diagnosis and differential diagnosis | 2017 | ^114^ |
| ^18^F-Alfatide | 25 | measurable lesions (n = 42) | diagnosis, therapeutic response | 2019 | ^144^ |
| ^18^F-Alfatide | 61 | esophageal squamous cell cancer | diagnosis and differential diagnosis | 2019 | ^119^ |
| ^18^F-Alfatide | 72 | adenocarcinoma (n = 37), squamous carcinoma (n = 35) | diagnosis and differential diagnosis | 2020 | ^117^ |
| ^18^F-Alfatide II | 14 | healthy volunteers (n = 5), brain metastases (n = 9) | safety, diagnosis | 2015 | ^131^ |
| ^18^F-Alfatide II | 30 | bone metastasis | diagnosis and differential diagnosis | 2015 | ^135^ |
| ^18^F-Alfatide II | 20 | inflammation [tuberculosis (n = 4), sarcoidosis (n = 3), common inflammation (n = 2)], lung cancer (n = 11) | diagnosis and differential diagnosis | 2018 | ^116^ |
| ^18^F-Alfatide II | 44 | suspected primary breast cancer | diagnosis and differential diagnosis | 2018 | ^80^ |
| ^68^Ga-NOTA-RGD | 43 | invasive ductal carcinoma | diagnosis and differential diagnosis | 2014 | ^81^ |
| ^68^Ga-NOTA-RGD | 3 | solitary pulmonary nodule | biodistribution, diagnosis | 2017 | ^62^ |
| ^68^Ga-DOTA-RGD | 5 | locally advanced breast carcinoma | biodistribution | 2015 | ^64^ |
| ^68^Ga-DOTA-RGD | 53 | breast cancer | diagnosis | 2015 | ^125^ |
| ^68^Ga-NODAGA-RGD | 1 | gastro-esophageal junction carcinoma | diagnosis | 2016 | ^118^ |
| ^68^Ga-NODAGA-RGD | 9 | hepatocellular carcinoma | biodistribution, diagnosis | 2016 | ^63^ |
| ^68^Ga-NODAGA-RGD | 10 | squamous cell carcinoma of head and neck | diagnosis, definition of tumor subvolume | 2020 | ^79^ |
| ^68^Ga-BBN-RGD | 18 | healthy volunteers (n = 5), prostate cancer (n = 13) | safety, diagnosis | 2017 | ^65^ |
| ^68^Ga-BBN-RGD | 22 | suspected breast cancer | diagnosis and differential diagnosis | 2018 | ^109^ |
| ^68^Ga-NOTA-3P-TATE-RGD | 44 | lung cancer [non-small cell lung cancer (n = 18), small cell lung cancer (n = 14)], neuroendocrine neoplasm [neuroendocrine tumor (n = 8), neuroendocrine carcinoma (n = 4)] | diagnosis and differential diagnosis | 2019 | ^85^ |
| ^68^Ga-DOTA-RGD2 | 31 | non-small cell lung cancer (n = 21), small cell lung cancer (n = 10) | diagnosis and differential diagnosis | 2017 | ^104^ |
| ^68^Ga-DOTA-RGD2 | 1 | chondroblastic osteosarcoma of the skull | diagnosis | 2017 | ^139^ |
| ^68^Ga-DOTA-RGD2 | 1 | papillary carcinoma thyroid | diagnosis, therapeutic response | 2018 | ^112^ |
| ^68^Ga-DOTA-RGD2 | 5 | locally advanced breast carcinoma | diagnosis | 2019 | ^124^ |
| ^68^Ga-DOTA-RGD2 | 44 | radioiodine-refractory differentiated thyroid cancer | diagnosis | 2020 | ^84^ |
| ^68^Ga-DOTA-RGD2 | 5 | healthy volunteers | safety, biodistribution | 2016 | ^70^ |
| ^68^Ga-DOTA-RGD2 | 10 | oral squamous cell carcinoma | safety, biodistribution, diagnosis | 2020 | ^69^ |
| ^68^Ga-NOTA-PRGD2 | 12 | newly diagnosed primary glioma | diagnosis and differential diagnosis | 2014 | ^107^ |
| ^68^Ga-NOTA-PRGD2 | 91 | suspected lung lesions | diagnosis and differential diagnosis | 2015 | ^93^ |
| ^68^Ga-NOTA-PRGD2 | 21 | uncommon meningioma (n = 5), high-grade glioma (n = 16) | diagnosis and differential diagnosis | 2018 | ^108^ |
| ^68^Ga-NODAGA-RGD2 | 5 | locally advanced breast cancer | biodistribution | 2016 | ^68^ |
